# Remodeling the tumor microenvironment via blockade of LAIR-1 and TGF-**β** signaling enables PD-L1–mediated tumor eradication

**DOI:** 10.1172/JCI155148

**Published:** 2022-04-15

**Authors:** Lucas A. Horn, Paul L. Chariou, Sofia R. Gameiro, Haiyan Qin, Masafumi Iida, Kristen Fousek, Thomas J. Meyer, Margaret Cam, Dallas Flies, Solomon Langermann, Jeffrey Schlom, Claudia Palena

**Affiliations:** 1 Laboratory of Tumor Immunology and Biology and; 2 CCR Collaborative Bioinformatics Resource (CCBR), Center for Cancer Research, National Cancer Institute (NCI), NIH, Bethesda, Maryland, USA.; 3 NextCure, Inc., Beltsville, Maryland, USA.

**Keywords:** Immunology, Cancer immunotherapy

## Abstract

Collagens in the extracellular matrix (ECM) provide a physical barrier to tumor immune infiltration, while also acting as a ligand for immune inhibitory receptors. Transforming growth factor-β (TGF-β) is a key contributor to shaping the ECM by stimulating the production and remodeling of collagens. TGF-β activation signatures and collagen-rich environments have both been associated with T cell exclusion and lack of responses to immunotherapy. Here, we describe the effect of targeting collagens that signal through the inhibitory leukocyte-associated immunoglobulin-like receptor-1 (LAIR-1) in combination with blockade of TGF-β and programmed cell death ligand 1 (PD-L1). This approach remodeled the tumor collagenous matrix, enhanced tumor infiltration and activation of CD8^+^ T cells, and repolarized suppressive macrophage populations, resulting in high cure rates and long-term tumor-specific protection across murine models of colon and mammary carcinoma. The results highlight the advantage of direct targeting of ECM components in combination with immune checkpoint blockade therapy.

## Graphical abstract

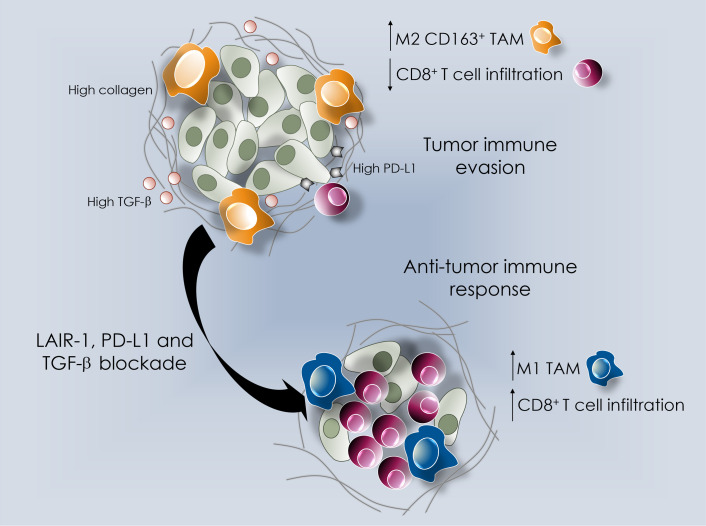

## Introduction

Immune checkpoint blockade (ICB) therapies targeting the programmed cell death 1 receptor (PD-1) or its ligand PD-L1 have shown unprecedented clinical benefit across various cancer types, yet durable responses have been achieved only in a limited subset of patients (1). Analyses of tumor biomarkers in clinical studies across various tumor types have identified factors that associate with response to ICB, including expression of PD-L1 on both tumor cells and tumor-infiltrating lymphocytes (TILs), tumor mutational burden, and TIL density, location, and activation (2). Beyond these factors, the presence of a collagen-dense extracellular matrix (ECM) and high numbers of immunosuppressive myeloid cell populations are increasingly being recognized as critical determinants of tumor responses to ICB therapy (3). Several tumor types that demonstrate poor responses to ICB, including pancreatic, breast, and colorectal cancers, are among those characterized by the presence of a collagen-dense ECM. Collagens can be secreted in the tumor microenvironment (TME) by cancer-associated fibroblasts (CAFs), cancer cells, and macrophages (4–6). Functioning as a physical barrier to immune cell infiltration into the tumor (7), a collagen-dense ECM has been shown to suppress antitumor immunity and to associate with PD-1/PD-L1–resistant tumors (8, 9).

A key contributor to the composition and abundance of the ECM is the cytokine transforming growth factor-β (TGF-β), which can be secreted in the TME by CAFs, myeloid cells, and cancer cells (10–13). TGF-β is a major regulator of the ECM homeostasis by not only stimulating the production of structural collagens by CAFs but also promoting the synthesis of enzymatic proteins that are involved in the crosslinking or degradation of collagens (14). Several studies have now linked a gene signature of TGF-β activation in tumors with T cell exclusion and lack of responses to ICB therapy (15–17).

In addition to providing a physical barrier to immune cell infiltration, collagens act as a ligand for a number of receptors expressed on tumor or immune cells. One such receptor is the leukocyte-associated immunoglobulin-like receptor-1 (LAIR-1, CD305), an immune checkpoint broadly expressed on immune cells, which delivers an inhibitory signal following binding to collagen-like domains. LAIR-1 signaling results in T cell exhaustion and suppression, and inhibition of natural killer (NK), monocyte, and dendritic cell (DC) activation and function (18, 19). While a soluble decoy receptor, LAIR-2, is expressed in humans and competes with LAIR-1 for binding to collagen-like domains, excess LAIR ligands in the tumor often result in an immunosuppressive environment (20).

We hypothesize that inhibition of immunosuppressive signals derived from a collagen-dense and TGF-β–enriched TME could synergize with PD-L1 therapy, resulting in enhanced antitumor responses. To test this hypothesis, an immunotherapy combination was evaluated in murine tumor models, consisting of co-inhibition of TGF-β, PD-L1, and LAIR-1 signaling. Colocalized, simultaneous inhibition of TGF-β and PD-L1 in the TME was achieved with the bifunctional fusion protein bintrafusp alfa, composed of 2 extracellular domains of the TGF-βRII fused to an anti–PD-L1 antibody (21, 22). This agent is currently being evaluated in multiple clinical studies (22), including in HPV-associated malignancies, for which an objective response rate of 30.5% was reported (23). Blockade of LAIR-1 signaling was achieved with a fusion protein consisting of 2 LAIR-2 molecules on an IgG1 backbone, NC410. A first-in-human clinical study of NC410 is currently ongoing (ClinicalTrials.gov NCT04408599).

Here we demonstrate that inhibition of TGF-β, PD-L1, and LAIR-1 was able to effectively control the growth of the collagen-rich murine MC38 colon and EMT6 breast carcinomas, resulting in tumor cures and long-term tumor-specific protection not achieved with individual compounds. This potent antitumor immune response took place in the context of tumor ECM remodeling, with decreased denatured collagen content, enhanced infiltration of activated CD8^+^ T cells, and remodeling of the myeloid tumor infiltrate.

## Results

### Blockade of LAIR-1 signaling synergizes with TGF-β and PD-L1 inhibition.

**Figure 1:**
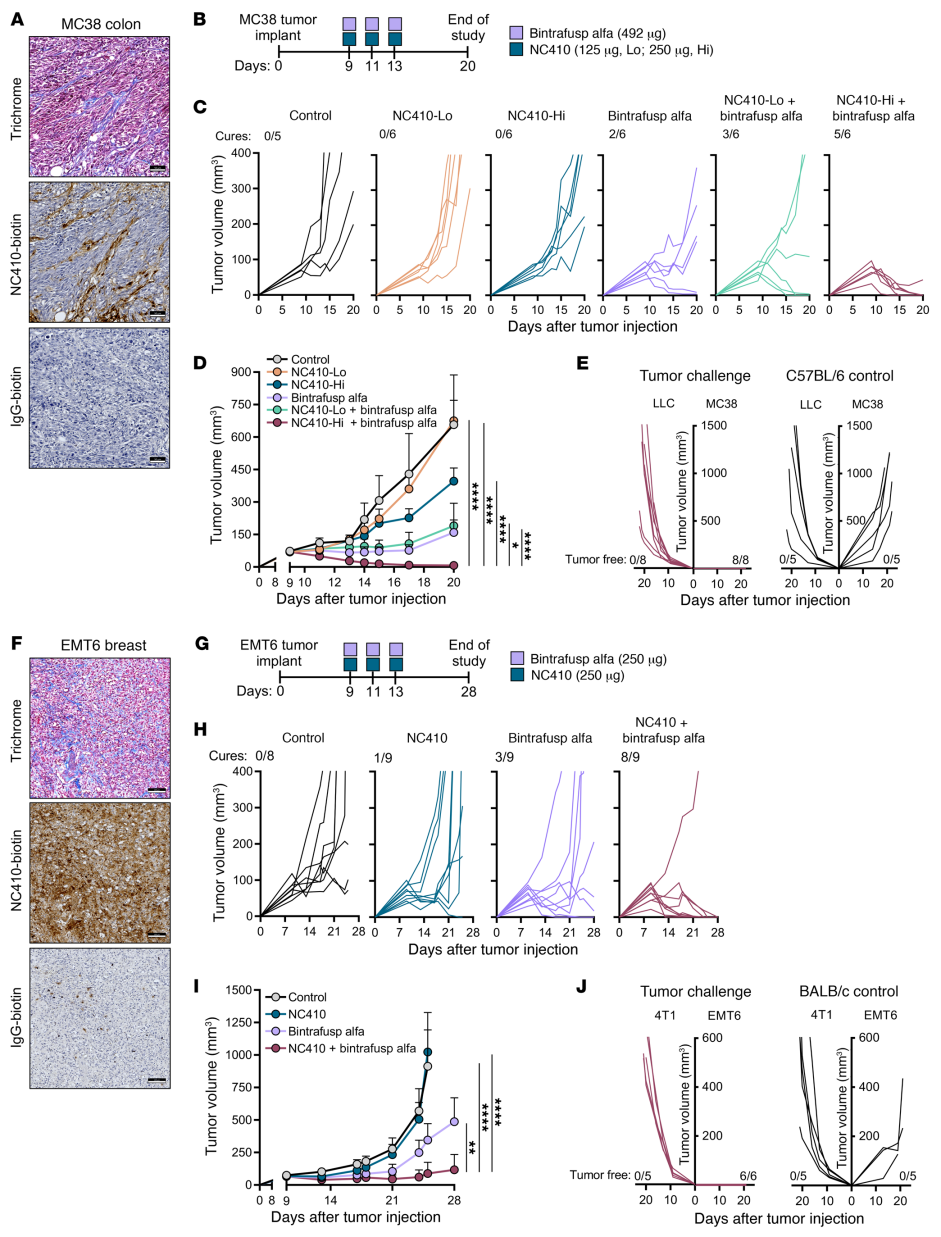
NC410 and bintrafusp alfa synergize for effective tumor control. (**A** and **F**) Representative images of MC38 and EMT6 tumors analyzed for collagen (trichrome staining), NC410-biotin, and control IgG-biotin staining. Scale bars: 50 μm (**A**) and 100 μm (**F**). (**B**) Treatment schedule for mice bearing MC38 tumors. Individual tumor growth and number of cures (**C**) and average tumor growth (**D**) are shown; *n =* 5 mice/group (control) or *n =* 6 (all other groups). Data are representative of 1 of 2 independent experiments. (**E**) Left: Cured mice were rechallenged s.c. with MC38 and LLC tumor cells. Right: Control C57BL/6 mice were injected s.c. with either MC38 or LLC tumor cells. Graphs show individual tumor growth and number of mice free of tumor. (**G**) Treatment schedule for mice bearing s.c. EMT6 tumors. Individual tumor growth and number of cures (**H**) and average tumor growth (**I**) are shown; *n =* 8 mice/group (control) or *n =* 9 (all other groups). (**J**) Left: Cured mice from the combination group were rechallenged s.c. with EMT6 and 4T1 tumor cells. Right: Control BALB/c mice were injected s.c. with either EMT6 or 4T1 tumor cells. Graph shows individual tumor growth. Error bars indicate SEM of biological replicates. *P ≤ 0.05; **P ≤ 0.01; ****P ≤ 0.0001 by 2-way ANOVA (**D** and **I**).

The therapeutic efficacy of NC410 plus bintrafusp alfa was first evaluated against the MC38 colon carcinoma model, which has been previously shown to be only partially responsive to anti–PD-L1 blockade therapy (21). Trichrome and immunohistochemistry (IHC) staining of MC38 tumors with biotinylated NC410 demonstrated high collagen content and binding of NC410 to collagen-rich areas, respectively, compared with IgG-biotin control (Figure 1A). Mice bearing subcutaneous (s.c.) MC38 tumors were administered intraperitoneal (i.p.) injections of NC410 at either a 125 μg (NC410-Lo) or 250 μg (NC410-Hi) dose alone or in combination with i.p. bintrafusp alfa (492 μg) on days 9, 11, and 13 after tumor injection (Figure 1B). While NC410 monotherapy had no effect on tumor growth, bintrafusp alfa monotherapy resulted in modest tumor control, with 2 out of 6 (33.3%) mice cured (Figure 1, C and D). In contrast, the combination of NC410 plus bintrafusp alfa led to complete tumor resolution in 3 out of 6 (50%) mice in the NC410-Lo and 5 out of 6 (83.3%) mice in the NC410-Hi combination groups (Figure 1, C and D). Tumor-free mice from the combination therapy groups were rechallenged after 12 weeks with s.c. MC38 cells, demonstrating successful rejection of the tumor cells in 8 out of 8 mice (Figure 1E). In contrast, cured mice were unable to reject an irrelevant tumor (LLC lung carcinoma), indicating that the protective memory was tumor specific. Naive mice used as controls developed both MC38 and LLC tumors (Figure 1E).

The effectiveness of the combination immunotherapy was also evaluated against the EMT6 breast carcinoma model, which has high collagen content and shows binding of NC410 to collagen-rich areas, compared with IgG-biotin control (Figure 1F). Mice bearing s.c. EMT6 tumors were administered i.p. injections of 250 μg NC410 and 250 μg bintrafusp alfa on days 9, 11, and 13 after tumor injection (Figure 1G). While NC410 or bintrafusp alfa monotherapies resulted in 1 out of 9 (11.1%) and 3 out of 9 (33.3%) cured mice, respectively, the combination of NC410 plus bintrafusp alfa led to robust tumor control, with 8 out of 9 (88.9%) mice showing complete tumor resolution (Figure 1, H and I). Tumor-free mice from the combination therapy group were also protected against EMT6 rechallenge, while rechallenge with irrelevant 4T1 breast cancer cells resulted in tumor growth (Figure 1J). As controls, naive mice implanted with either EMT6 or 4T1 cells developed tumors (Figure 1J). The potential toxicity of this immunotherapy was evaluated by using the same treatment schedule as in Figure 1B to deliver 250 μg NC410 plus 492 μg bintrafusp alfa to nontumor- and MC38 tumor–bearing C57BL/6 mice. Based on animal weight, complete blood count (CBC), serum chemistry, and organ histopathology, no toxicities were observed in any of the treated mice relative to the controls (Supplemental Figure 1, A–D; supplemental material available online with this article; https://doi.org/10.1172/JCI155148DS1).

**Figure 2:**
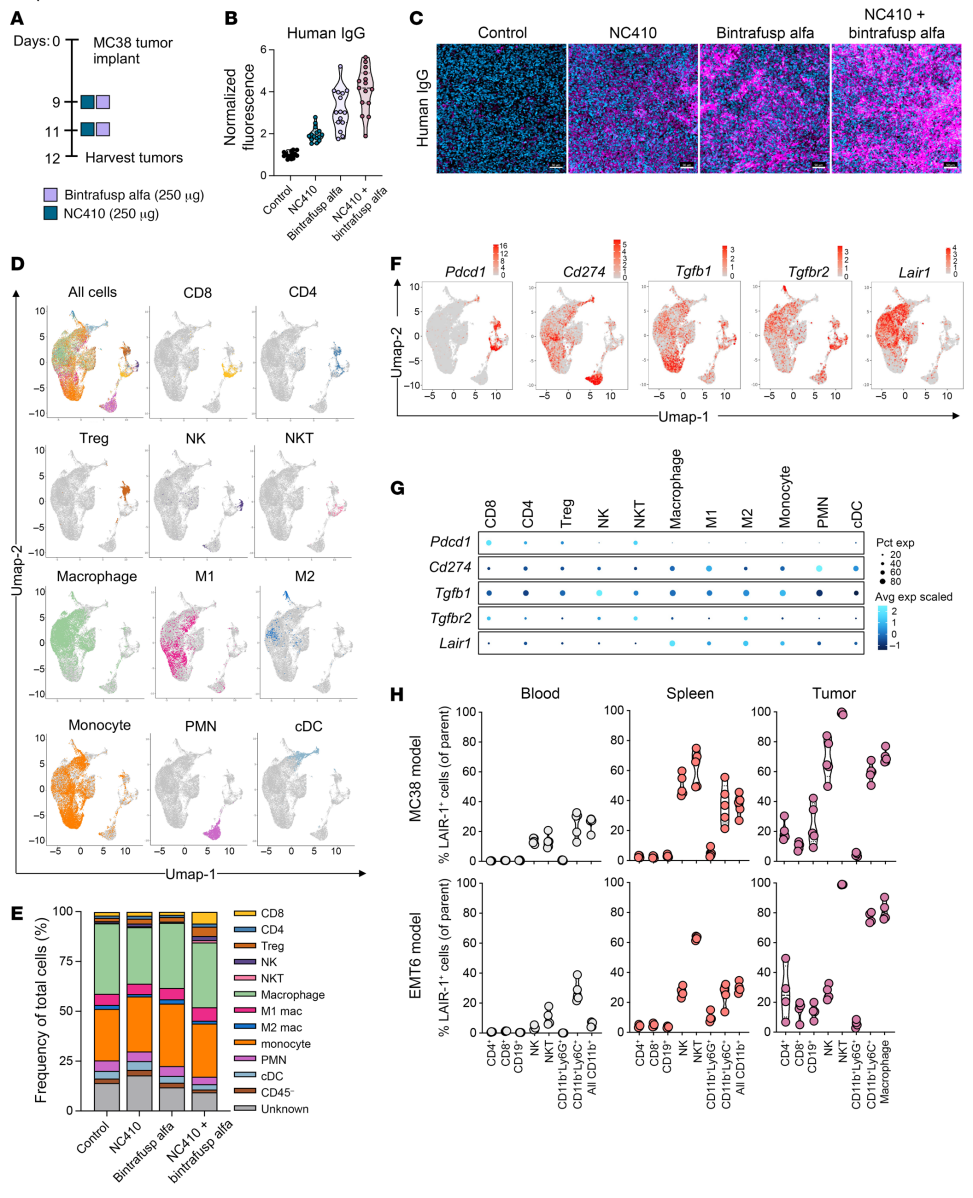
Expression of target molecules and treatment effect on tumor immune infiltrates. (**A**) Treatment schedule for mice bearing MC38 tumors. (**B**) Immunofluorescence-based analysis of harvested tumors for human IgG, indicating presence of therapeutic agents. Graph shows the fluorescence signal across 15 regions of interest (ROIs) randomly selected within each tumor section, *n =* 2–3 tumors per treatment group, normalized to the average signal in the control group. (**C**) Representative images of MC38 tumors. Scale bar: 50 μm. (**D**) scRNA-seq profiling of tumor-infiltrating CD45^+^ cells isolated from tumors treated as indicated in **A**. UMAP plots for all treatment groups combined and analyzed as described in the Methods section, showing selected identified immune cell subset clusters with events colored according to cell type. (**E**) Frequency of selected cell subsets identified by scRNA-seq analysis from MC38 tumors treated as indicated in **A**. (**F** and **G**) Expression of selected genes of relevance by scRNA-seq in single-color UMAP plots (**F**) or in bubble plot representation across selected immune cell subset clusters. Bubble size shows percentage of cells expressing the indicated gene; color intensity represents scaled expression levels. Data from scRNA-seq analysis are from a single experiment. (**H**) Flow cytometry analysis of LAIR-1 expression on indicated immune cell subsets in the blood, spleen, and tumors from MC38 and EMT6 tumor–bearing mice; *n =* 5 mice/group (MC38), *n =* 4/group (EMT6). Tissues for analysis were collected on day 12 prior to any treatment. For violin plots, dashed line displays the median and dotted lines display quartiles. Data from MC38 spleen and tumor flow cytometry are representative of 1 of 2 independent experiments.

To conduct mechanistic studies and owing to the rapid and effective resolution of tumors treated with the optimized regimen described above, MC38 tumor–bearing mice were treated with only 2 doses of NC410 and bintrafusp alfa on days 9 and 11; tumors were collected early (day 12) for analyses (Figure 2A). Both NC410 and bintrafusp alfa contain human IgG1 domains; therefore, the presence of these agents at the tumor site was determined with the use of anti–human IgG staining. MC38 tumors treated with NC410, bintrafusp alfa, and NC410 plus bintrafusp alfa showed approximately 1.9-fold, 3.1-fold, and 3.9-fold higher, respectively, anti–human IgG signal compared with baseline levels in control tumors (Figure 2, B and C). In order to understand which immune cell subsets are affected by the combination therapy, analysis of the tumor immune transcriptome was performed by single-cell RNA sequencing (scRNA-seq) of CD45^+^ tumor-infiltrating cells pooled from MC38 control and treated tumors. All sequenced cells were clustered into unbiased cell type classifications using the Seurat single-cell analysis R package and visualized with uniform manifold approximation and projection (UMAP). Previously published gene sets (24) were optimized for identification of murine immune cell subtypes in MC38 tumors (Supplemental Table 1 and Supplemental Figure 2A). A total of 41 distinct cell clusters were identified by dimension reduction analysis and named according to the most abundant immune cell subset identified in the cluster (Supplemental Figure 2, A and B). Distribution of immune cell subsets across clusters was visualized with UMAP, including CD8^+^, CD4^+^, T regulatory (Treg) cells, NK cells, NK T (NKT) cells, macrophages, M1 and M2 macrophages, monocytes, polymorphonuclear cells (PMNs), and conventional DCs (cDCs) (Figure 2D). Shown in Figure 2E and Supplemental Table 2 are the frequencies and numbers of selected subsets across treatment groups.

Initial transcriptomic analysis for the presence of NC410 and bintrafusp alfa targets on MC38 tumor–infiltrating immune cells within all treatment groups (Figure 2, F and G) revealed high expression of *Pdcd1* (encoding PD-1) in T cells, mainly CD8^+^, and NKT cells, while the gene encoding PD-L1 (*Cd274*) was expressed in most immune cells, particularly PMNs, M1 macrophages, and cDCs. *Tgfb1* was also found across all cell clusters, with higher expression observed in NK cells and monocytes, while *Tgfbr2* was predominantly expressed in CD8^+^ T cells, NKT cells, and M2 macrophages. Expression of *Lair1* was found mostly across myeloid cell clusters, with higher expression in M2 versus M1 macrophages. This interesting pattern of expression was validated at the protein level by flow cytometry analysis on multiple immune cell subsets in the blood, spleen, and tumor of both MC38 and EMT6 tumor–bearing mice (Figure 2H). Overall, CD4^+^, CD8^+^, CD19^+^, and CD11b^+^Ly6G^+^ subsets had fewer LAIR-1^+^ cells, while NK cells, NKT cells, CD11b^+^Ly6C^hi^ cells, and CD11b^+^F4/80^hi^ macrophages had higher percentages of LAIR-1^+^ cells, particularly at the tumor site compared with the spleen and blood (Figure 2H). These data validated PD-1/PD-L1, TGF-β, and LAIR-1 as potentially actionable targets in MC38 tumors.

### Combination therapy increases tumor infiltration with activated CD8^+^ T cells.

**Figure 3:**
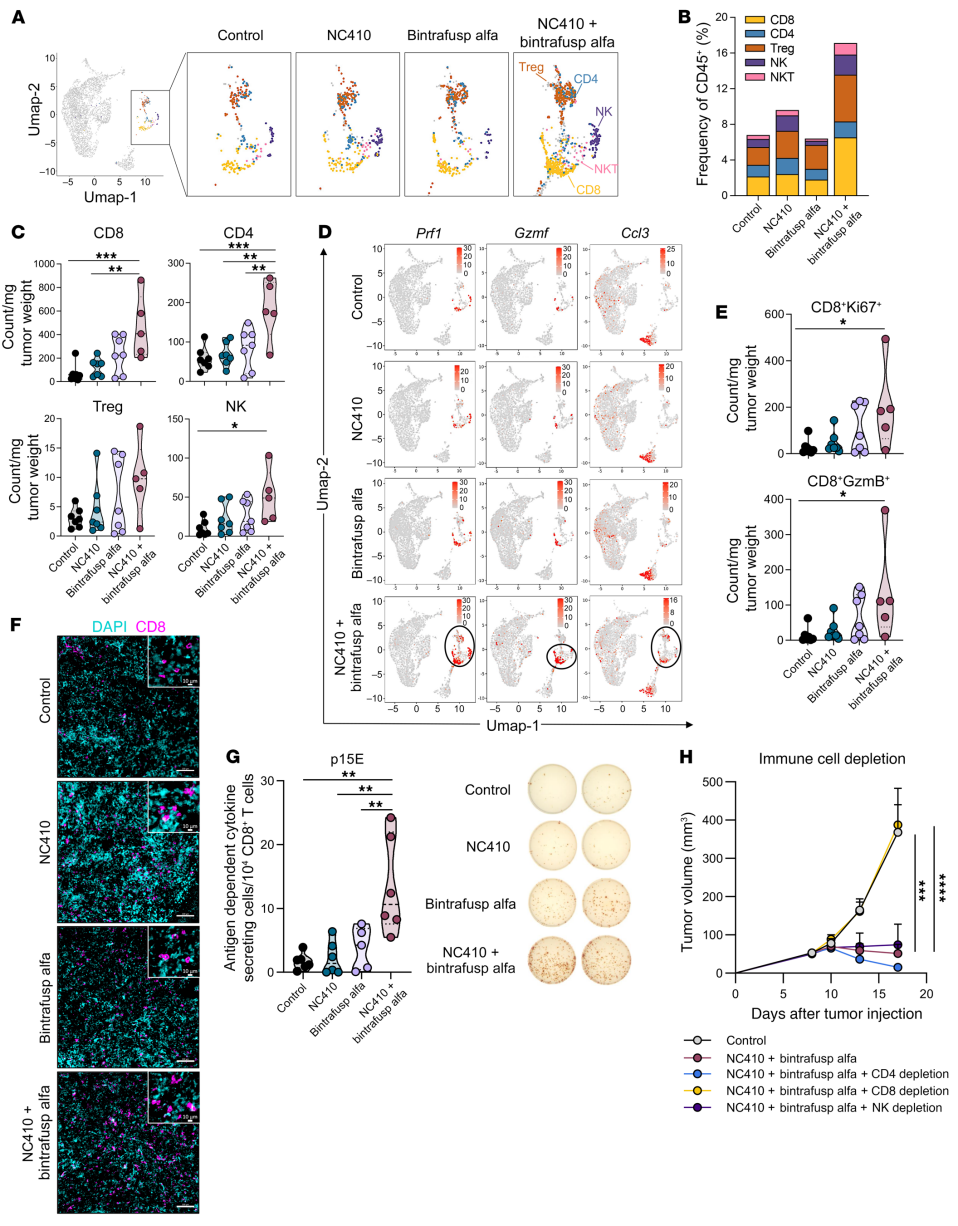
Combination therapy increases infiltration with activated CD8^+^ T cells. (**A**) UMAP plots showing CD4^+^ and CD8^+^ T cells, T regulatory (Treg) cells, NK and NKT cell clusters as identified by scRNA-seq analysis from MC38 tumors treated as in Figure 2A. (**B**) Frequency of indicated immune cell subsets, as a percentage of total CD45^+^ cells. (**C**) Flow cytometry analysis of indicated immune infiltrating cells or (**E**) analysis of CD8^+^ T cells for expression of Ki67 or granzyme B (GzmB) in MC38 tumors collected on day 17 following treatment with NC410 (250 μg) and/or bintrafusp alfa (250 μg) on days 9, 11, and 13. Graphs show the number of cells per mg tumor weight; *n =* 7 (control, NC410, bintrafusp alfa), *n =* 5 (NC410 + bintrafusp alfa). (**D**) UMAP plots showing expression of selected genes by scRNA-seq. (**F**) Representative images of CD8^+^ T cell infiltrates (magenta) in MC38 tumors treated as indicated in Figure 2A. DAPI (cyan) was used as a nuclear stain. Scale bars: 100 μm and 10 μm (insets). (**G**) IFN-γ ELISPOT analysis of spleens from MC38 tumor–bearing mice treated as indicated, against the p15E tumor antigen; *n =* 6/group. Representative images of well signals from 2 individuals per group are displayed. (**H**) Average tumor growth of MC38 tumors untreated or treated with NC410 plus bintrafusp alfa with or without depleting antibodies for CD4^+^, CD8^+^, or NK cells; *n =* 6 in the NK depletion group; *n =* 7 in all other groups. For violin plots, dashed line displays the median and dotted lines display quartiles. Error bars indicate SEM of biological replicates. *P ≤ 0.05; **P ≤ 0.01; ***P ≤ 0.001; ****P ≤ 0.0001 by 1-way ANOVA followed by Tukey’s post hoc test in **C**, **E**, and **G** and 2-way ANOVA in **H**.

Transcriptomic analysis of MC38 tumor immune-infiltrating cells revealed increased frequency of T and NK cell subsets in NC410 plus bintrafusp alfa–treated tumors, compared with all other groups, with an increase in CD8^+^ T cells (3.0-fold), NK cells (2.5-fold), NKT cells (2.9-fold), and Treg cells (2.6-fold) in the combination compared with the control group (Figure 3, A and B). To extend these observations at the protein level, flow cytometry analysis of immune-infiltrating cells was performed on control and agent-treated MC38 tumors. Significantly higher numbers of CD8^+^ T cells, CD4^+^ T cells, and NK cells were detected in MC38 tumors treated with NC410 plus bintrafusp alfa, compared with the control (Figure 3C). scRNA-seq analysis of CD8^+^ T cells showed upregulation of genes encoding the cytolytic proteins perforin (*Prf1*) and granzyme F (*Gzmf*), and the T cell chemoattractant chemokine *Ccl3* in the combination group (Figure 3D), indicative of a highly activated CD8^+^ T cell phenotype. Similar results were observed via flow cytometry analysis (Figure 3E) showing higher numbers of proliferative (Ki67^+^) and granzyme B–positive (GzmB^+^) CD8^+^ T cells in NC410 plus bintrafusp alfa–treated tumors compared with the control. Using immunofluorescence-based staining, the greatest number and density of CD8^+^ T cells was found in MC38 tumors treated with the combination therapy (Figure 3F), with CD8^+^ T cells homogeneously distributed within the tumor parenchyma and not excluded to the tumor/stroma border. Furthermore, an ELISPOT assay was performed to comparatively evaluate the level of T cells specific for a representative MC38 tumor–associated antigen, p15E, in the spleen. While only low levels of p15E-specific T cells were detected in untreated mice or mice treated with NC410 or bintrafusp alfa monotherapy, a marked and significant increase was observed in the combination-treated mice compared with the other groups (Figure 3G). To confirm that CD8^+^ T cells were essential for the efficacy of the combination therapy, a depletion study was conducted whereby CD8^+^ T cell depletion completely abrogated the antitumor effect of the NC410 plus bintrafusp alfa therapy (Figure 3H). Although NK cells and CD4^+^ T cells also increased with combination treatment, the depletion of NK cells did not have an effect, while CD4^+^ T cell depletion increased rather than decreased the antitumor efficacy of the combination (Figure 3H), an effect presumably due to the depletion of Tregs achieved via CD4^+^ T cell depletion. In addition to T cell activation, transcriptomic analysis revealed numerous activated pathways in NK cells from combination-treated compared with control tumors, including pathways involved in positive regulation of interferon-γ (IFN-γ) production, type 1 IFN signaling, and innate immune response (Supplemental Figure 3 and Supplemental Table 3). Collectively, these data indicated that NC410 plus bintrafusp alfa therapy increases the frequency, activation, and cytotoxic activity of CD8^+^ T cells in the TME, leading to effective tumor resolution.

### NC410 and bintrafusp alfa synergize to remodel the myeloid cell composition of the TME.

**Figure 4:**
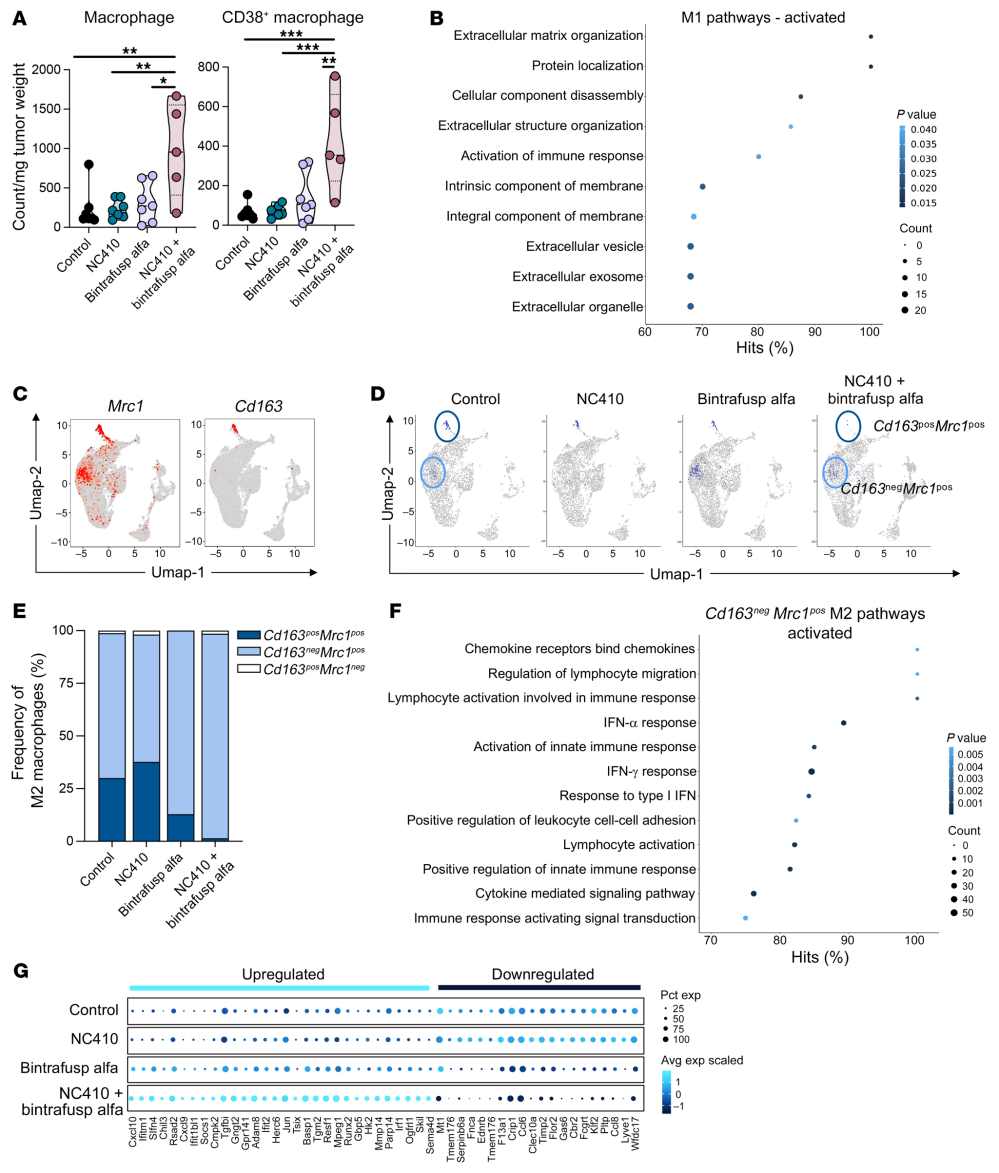
NC410 plus bintrafusp alfa reduces tumor infiltration with tumor-associated M2 macrophages. (**A**) Flow cytometry analysis of total macrophages and CD38^+^ macrophages in MC38 tumors collected on day 17 following treatment with NC410 (250 μg) and/or bintrafusp alfa (250 μg) on days 9, 11, and 13. Graphs show the number of cells per mg tumor weight; *n =* 7 (control, NC410, bintrafusp alfa), *n =* 5 (NC410 + bintrafusp alfa). For violin plots, dashed line displays the median and dotted lines display quartiles. (**B**) Top 10 activated GO gene pathways in M1 clusters identified by scRNA-seq in the NC410 plus bintrafusp alfa versus the control group. UMAP plots showing (**C**) expression of *Mrc1* and *Cd163* genes used to identify M2 cell clusters by scRNA-seq, and (**D**) variations in their expression across treatment groups. (**E**) Frequency of subpopulations of M2 macrophages according to their expression of *Cd163* and *Mrc1*. (**F**) Selected activated GO/REACTOME/KEGG/HALLMARK gene pathways in *Cd163*^neg^ *Mrc1*^pos^ M2 clusters identified by scRNA-seq in the NC410 plus bintrafusp alfa versus the control group. (**G**) Bubble plot representation of the top 30 upregulated and top 20 downregulated genes (logFC ≥ 0.25 or ≤ –0.25 and *P* value ≤ 0.05) in *Cd163*^neg^ *Mrc1*^pos^ M2 clusters from the NC410 plus bintrafusp alfa group versus the control group. Bubble size shows percentage of cells expressing the indicated gene; color intensity represents scaled expression levels. *P ≤ 0.05; **P ≤ 0.01; ***P ≤ 0.001 by 1-way ANOVA followed by Tukey’s post hoc test in **A**.

Due to the high expression of *Tgfbr2* and LAIR-1 in MC38 tumor–infiltrating macrophages, we hypothesized that blockade of LAIR-1 signaling via NC410 could synergize with TGF-β inhibition mediated by bintrafusp alfa to remodel the myeloid cell composition of the TME. Flow cytometry analysis revealed a significant increase in the number of total macrophages (CD45^+^CD11b^+^F4/80^hi^) and specifically CD38^+^ M1 macrophages (CD45^+^CD11b^+^F4/80^hi^CD38^+^) in MC38 tumors treated with NC410 plus bintrafusp alfa versus any other group (Figure 4A). Interestingly, when scRNA-seq data were interrogated to evaluate the transcriptomic composition of the M1 cell cluster, Gene Ontology (GO) analysis indicated a significant activation of several pathways related to the reorganization and structure of the ECM (Figure 4B). Changes in the composition of the M2 macrophage cluster were also evaluated via scRNA-seq analysis. Module scoring identification of M2 macrophages utilized the positive expression of the mannose receptor C-type 1 (*Mrc1*) and the scavenger receptor CD163 (*Cd163*), resulting in 2 distinct M2 macrophage clusters — *Cd163*^pos^ *Mrc1*^pos^ (*Cd163*^pos^ M2) and *Cd163*^neg^ *Mrc1*^pos^ (*Cd163*^neg^ M2) cells (Figure 4, C and D). While control tumors presented with an almost 1:4 ratio of *Cd163*^pos^ to *Cd163*^neg^ M2 macrophages (Figure 4E), NC410 plus bintrafusp alfa therapy almost completely abrogated the *Cd163*^pos^ M2 cluster (Figure 4, D and E). Differentially expressed gene and GO analyses of the remaining *Cd163*^neg^ M2 cells in the combination-treated tumors (Figure 4F and Supplemental Table 4) also showed significant activation of pathways involved in lymphocyte activation and migration, activation of innate immune response, and IFN-α and IFN-γ responses. Numerous genes were significantly upregulated in the *Cd163*^neg^ M2 cluster in MC38 tumors treated with the combination therapy versus control (Figure 4G), including the genes encoding the T cell chemoattractant chemokines CXCL10 and CXCL9, which are more synonymous with an M1 phenotype. In addition, several genes encoding ECM organization and remodeling were either upregulated (*Adam8*, *Tgm2*, *Mmp14*, and *Tfgb1*) or downregulated (*Timp2*). Collectively, these analyses provided evidence that NC410 plus bintrafusp alfa therapy is able to remodel the composition and transcriptional plasticity of the macrophage compartment in MC38 tumors, including depleting *Cd163*^pos^ M2 clusters and inducing a more M1-like immune-favorable environment.

### NC410 plus bintrafusp alfa therapy remodels the ECM.

**Figure 5:**
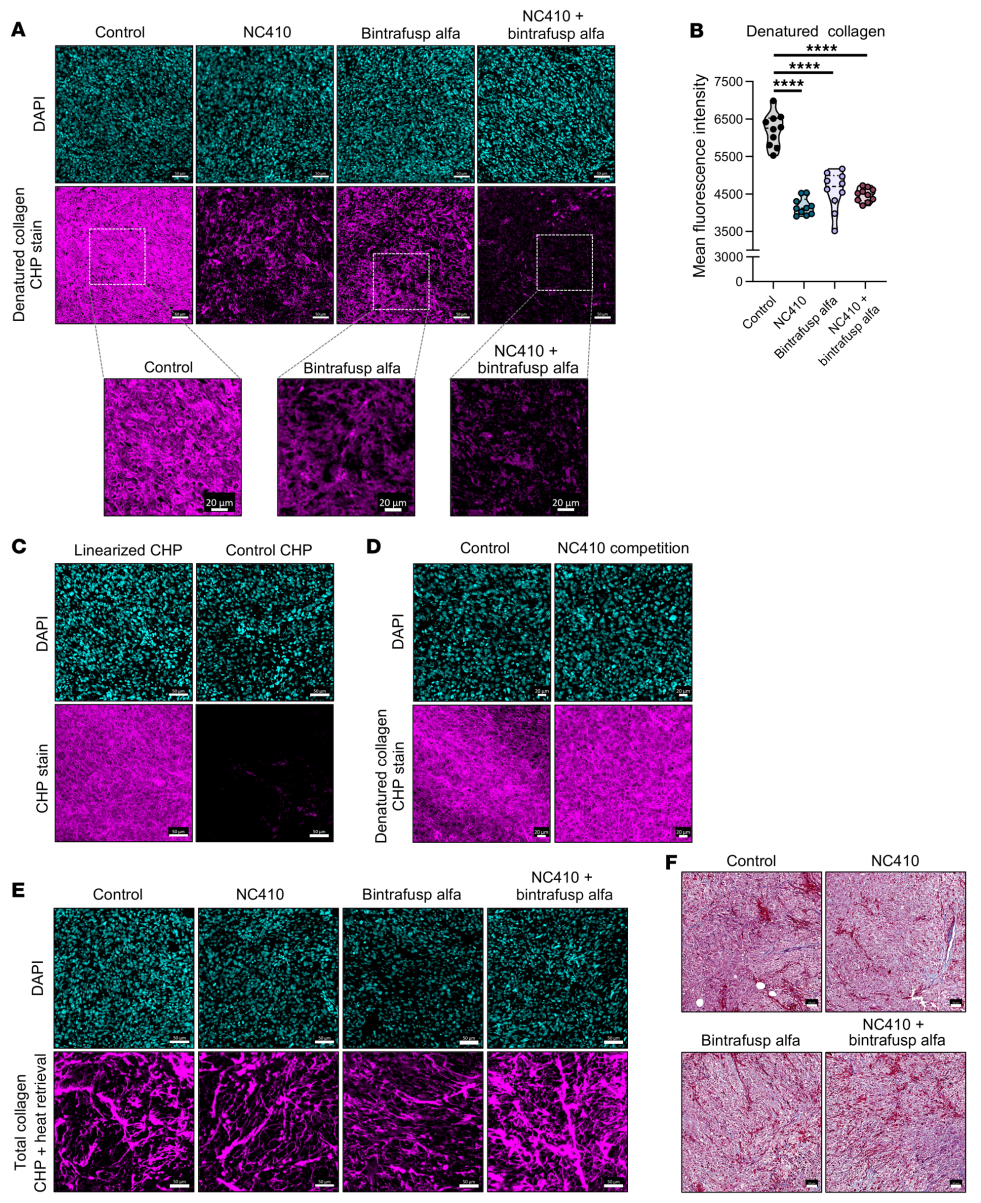
Remodeling of collagen in tumors treated with NC410 plus bintrafusp alfa therapy. (**A**) Representative images of immunofluorescence-based staining of denatured collagen utilizing a linearized collagen hybridizing peptide (CHP, magenta) in MC38 tumors collected as indicated in Figure 2A. White dash–outlined squares identify magnified regions in bottom images. (**B**) Mean fluorescence intensity value of denatured collagen across treatment groups; 10 regions of interest (ROIs) randomly selected within each tumor section; *n =* 3 tumors per treatment group. For violin plots, dashed line displays the median and dotted lines display quartiles. (**C**) Staining of denatured collagen in control MC38 tumors utilizing linearized CHP peptide or nonlinearized CHP as a negative control. (**D**) Staining of denatured collagen in control MC38 tumors in the absence or presence of NC410 to rule out competition for binding to collagens. (**E**) Total collagen content measured with CHP staining after heat retrieval in MC38 tumors collected 1 day following the second dose of treatments, as indicated in Figure 2A. DAPI staining of nuclei (cyan) is shown. (**F**) Representative images of MC38 tumors treated with control, NC410, bintrafusp alfa, and NC410 plus bintrafusp alfa analyzed for collagen expression by trichrome staining. Scale bars: 20 μm (**A**, bottom panels, and **D**), 50 μm (**A**, **C**, and **E**), and 100 μm (**F**). ****P ≤ 0.0001 by 1-way ANOVA followed by Tukey’s post hoc test in **B**.

The activation of several pathways related to the remodeling and structure of the ECM in tumor-infiltrating macrophages in the combination treatment group led us to investigate possible changes in the composition of the collagenous component of the ECM. For these studies, a fluorescently conjugated collagen-hybridizing peptide (CHP) was utilized, which specifically binds to denatured collagen strands by reforming a triple helix characteristic of collagenous proteins. Following staining with the linearized CHP peptide, strong fluorescence signal was detected in control MC38 tumors, which presented as a nonfibrillar structure with a lattice pattern (Figure 5A). In contrast, tumors corresponding to the NC410 and the combination NC410 plus bintrafusp alfa and, to a lesser extent, the bintrafusp alfa group showed significantly decreased signal, indicating the loss of denatured collagen content (Figure 5, A and B). As a negative control, staining with the nonlinearized CHP peptide (control CHP) was performed, which demonstrated no binding to control MC38 tumor tissues (Figure 5C). To rule out that binding of the CHP peptide was being precluded by binding of NC410 to collagens, a competition assay was also performed by incubating tumor slides with NC410 protein prior to staining with the CHP peptide; as shown in Figure 5D, no competition was observed. We next investigated whether total collagen content was also being altered in the treated tumors. Heat-induced retrieval at 95°C was performed to denature all collagens prior to CHP stain; unlike with denatured collagen, the total collagenous content of the tumors, mostly constituted of highly organized fibrillar structures, was not altered with treatment, although a trend was observed toward a higher fibrillar organization in the combination-treated MC38 tumors (Figure 5E). Similar results were observed when trichrome staining was utilized to assess collagens in tumor tissues (Figure 5F), with no differences in collagen amount or overall morphology observed across groups. These results demonstrated a restructuring of the collagenous matrix in tumors treated with the combination therapy, with almost complete depletion of denatured collagens.

### Blockade of LAIR-1 ligands, PD-L1, and TGF-β is indispensable for effective tumor control.

**Figure 6:**
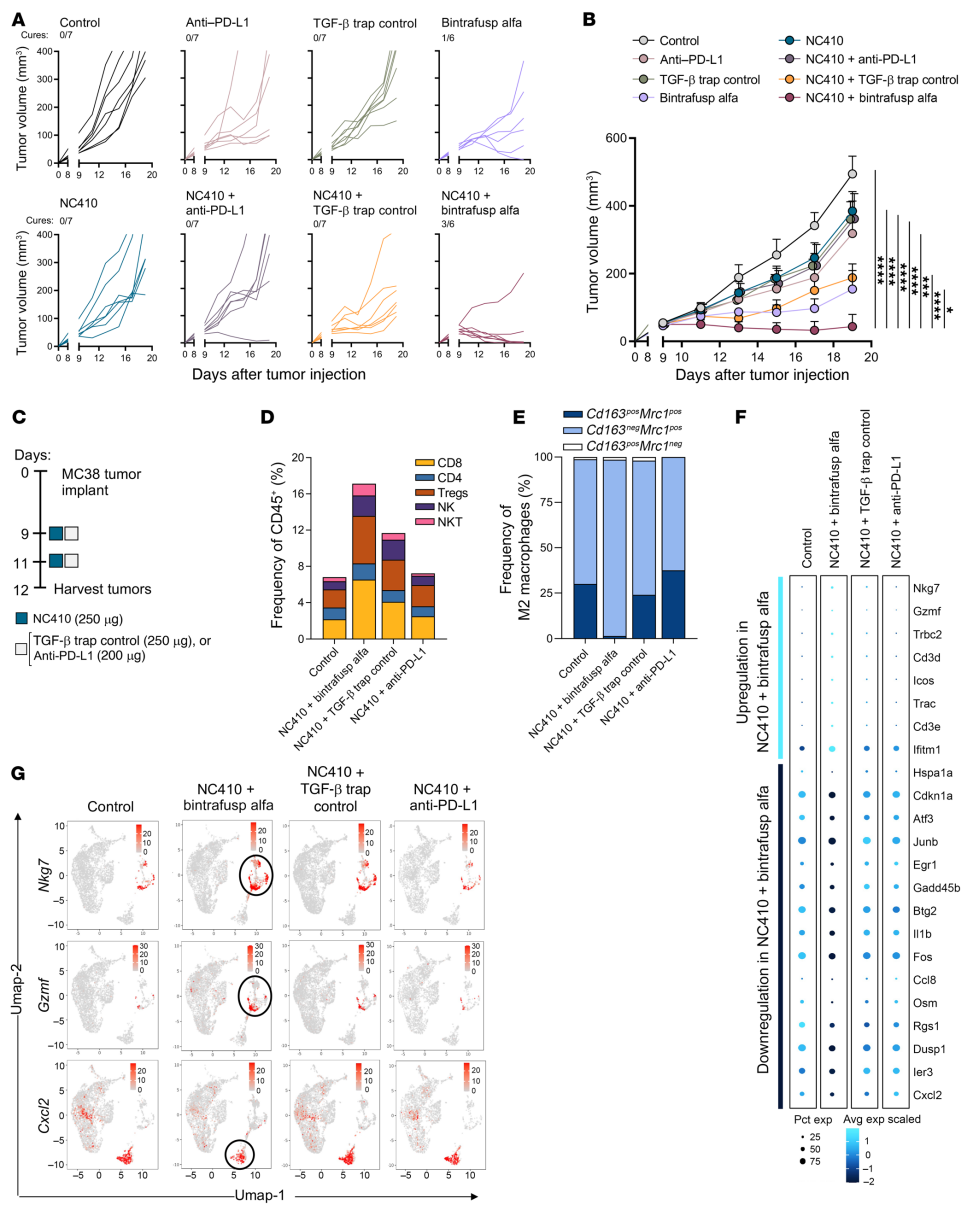
Inhibition of TGF-β and PD-L1 are both required for optimal tumor control in combination with NC410. MC38 tumor–bearing mice were administered indicated doses of NC410, bintrafusp alfa, anti–PD-L1, or TGF-β trap control on days 9, 11, and 13 after tumor injection. Graphs show (**A**) individual tumor growth and number of cures in each group, and (**B**) average tumor growth; *n =* 6 mice/group (bintrafusp alfa, NC410 + bintrafusp alfa) or *n =* 7 (control, anti–PD-L1, TGF-β trap control, NC410, NC410 + anti–PD-L1, NC410 + TGF-β trap control). Data are representative of 1 of 2 independent experiments. Error bars indicate SEM of biological replicates. *P ≤ 0.05; ***P ≤ 0.001; ****P ≤ 0.0001 by 2-way ANOVA. (**C**) Treatment schedule of indicated therapeutic agents; CD45^+^ cells isolated from MC38 tumors collected on day 12 were used for scRNA-seq analysis. (**D**) Frequency of effector CD4^+^, CD8^+^, T regulatory (Treg) cells, NK and NKT cell clusters as determined by scRNA-seq, shown as a percentage of total CD45^+^ cells. (**E**) Frequency of subpopulations of M2 macrophages according to their expression of *Cd163* and *Mrc1*. (**F**). Bubble plot representation of all genes differentially expressed (logFC ≥ 0.25 or ≤ –0.25 and *P* value ≤ 0.05) in total CD45^+^ cells from the NC410 plus bintrafusp alfa group versus all other groups. Bubble size shows percentage of cells expressing the indicated gene; color intensity represents scaled expression levels. (**G**) UMAP plots showing expression of selected genes by scRNA-seq analysis on CD45^+^ cells in each treatment group. Data from scRNA-seq analysis are from a single experiment; control and NC410 plus bintrafusp alfa groups from Figures 2–4 are shown for comparison.

To determine whether blockade of all 3 pathways (LAIR-1, PD-L1, and TGF-β) would be required to achieve optimal antitumor efficacy, bintrafusp alfa, anti–PD-L1, and a mutant version of bintrafusp alfa (designated as TGF-β trap control), which has no binding to PD-L1 while still sequestering TGF-β, were utilized as monotherapy or in combination with NC410 to comparatively treat MC38 tumor–bearing mice. As in previous experiments, agents were administered on days 9, 11, and 13 after tumor injection. Bintrafusp alfa and the TGF-β trap control agent were administered at 250 μg, and anti–PD-L1 was administered at an equimolar ratio (200 μg). As shown in Figure 6, A and B, the robust antitumor effect of the combination NC410 plus bintrafusp alfa was not achieved with any of the monotherapy or combinations of NC410 with anti–PD-L1 or the TGF-β trap control. Of note, the combination of NC410 plus TGF-β trap control resulted in relatively modest tumor delay, similar to that of bintrafusp alfa monotherapy. These data indicated that simultaneous blockade of LAIR-1, PD-1/PD-L1, and TGF-β pathways is necessary for optimal tumor control.

To further determine the mechanism of action, single-cell transcriptomic analysis from CD45^+^ immune-infiltrating cells collected from tumors on day 12 after 2 doses of agents and prior to tumor cures (Figure 6C) was used to compare the NC410 combination groups with anti–PD-L1 or the TGF-β trap control devoid of PD-L1 binding. These analyses were directly compared to the control and NC410 plus bintrafusp alfa data described in Figures 2–4. Interrogation of immune cell clusters identified by scRNA-seq analysis showed increased frequency of both CD8^+^ T cells (1.9-fold) and NK cells (2.4-fold) in the NC410 plus TGF-β trap control group, which were relatively less pronounced than those observed with NC410 plus bintrafusp alfa therapy (Figure 6D). Comparison of M2 macrophage clusters (Figure 6E), however, revealed that depletion of the *Cd163*^pos^ M2 cluster only occurred in the NC410 plus bintrafusp alfa combination group. While genes encoding some immune activation markers such as the cytolytic proteins NK cell granule protein 7 (*Nkg7*) and GzmF were upregulated in total CD45^+^ cells from the NC410 plus TGF-β trap control group compared with the control (Figure 6, F and G), expression of these markers was highest in the NC410 plus bintrafusp alfa combination. Similarly, PMNs in the NC410 plus bintrafusp alfa group had significantly lower expression of *Cxcl2* (Figure 6, F and G), which encodes a chemokine known to attract PMNs and myeloid-derived suppressor cells and to modulate tumor cell plasticity (25, 26), compared with all other groups. These results suggested that while NC410 plus TGF-β sequestration mediated by the TGF-β trap control agent can promote increased frequencies of NK and CD8^+^ T cells, blockade of LAIR-1, PD-1/PD-L1, and TGF-β pathways synergizes for optimal immune cell activation and repolarization of macrophages in the TME.

### NC410 alters the phenotype of alternatively activated (M2-like) human macrophages in vitro.

**Figure 7:**
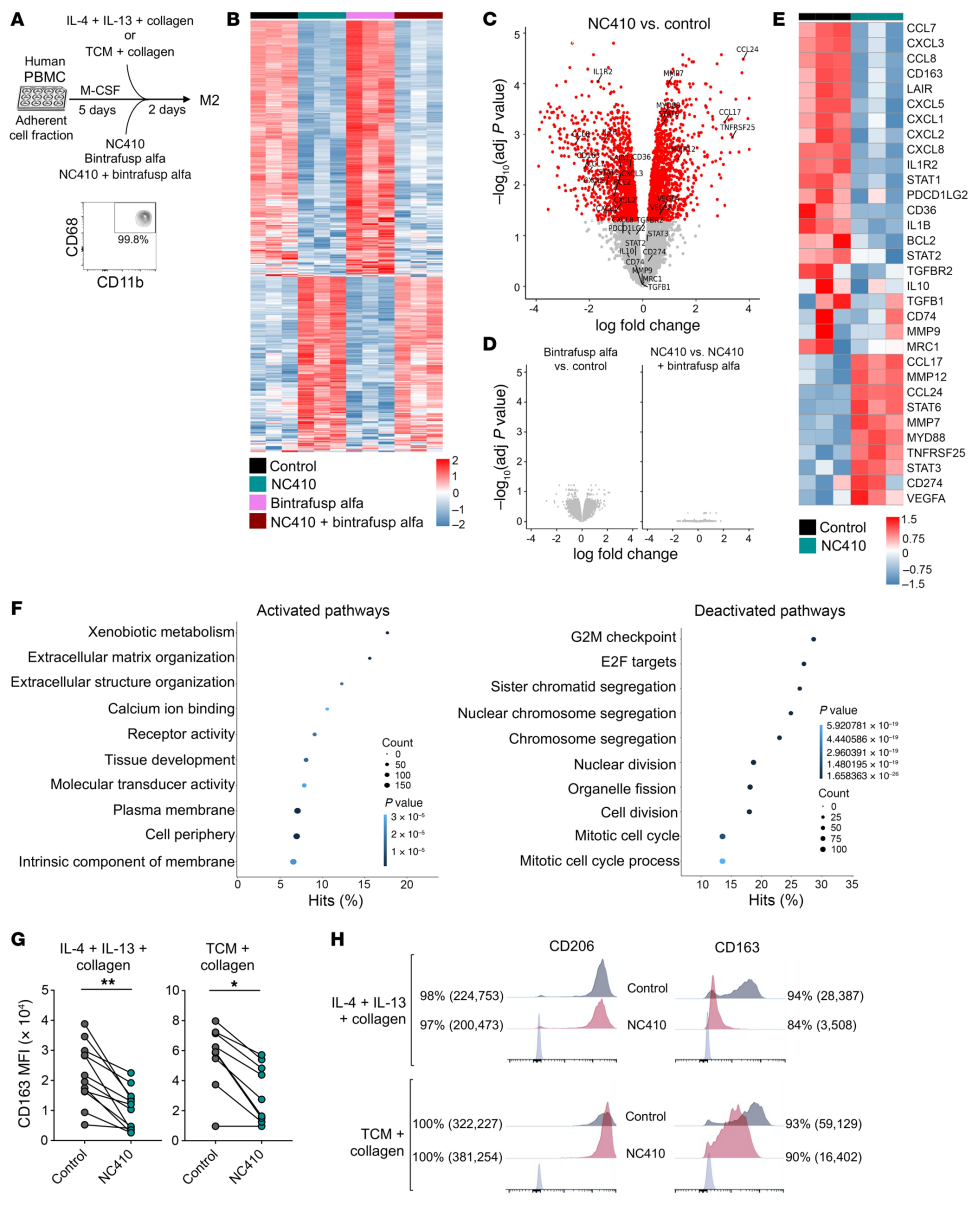
NC410 alters the M2 polarization phenotype of human macrophages in vitro. (**A**) Schematic detailing human macrophage polarization process and purity as determined by flow cytometry via CD68^+^/CD11b^+^ staining. (**B**) Gene expression heatmap based on RNA-seq analysis depicting the top 500 genes differentially expressed by variance in M2-like polarized macrophages across all treatment groups; (*n =* 3 donors/group). Volcano plots of differentially expressed genes between (**C**) NC410-treated and control groups, (**D**) bintrafusp alfa versus control and NC410 versus NC410 plus bintrafusp alfa groups; red dots indicate genes with an adjusted *P* value ≤ 0.05; genes related to M1/M2 macrophage polarization are indicated. (**E**) Gene expression heatmap depicting selected M1/M2 macrophage polarization genes in control and NC410-treated M2-like human macrophages; shown at the bottom is the *z*-score scale. (**F**) Top 10 significantly activated (left panel) and deactivated (right panel) GO/REACTOME/KEGG/HALLMARK gene pathways in NC410-treated versus control M2-like human macrophages. (**G**) Flow cytometry data depicting the mean fluorescence intensity (MFI) of CD163 expression on M2-like macrophages prepared from PBMCs from healthy donors via culture in the presence of IL-4, IL-13, and collagen (*n =* 12 donors) or a mix of tumor-conditioned media (TCM) and collagen (*n =* 9 donors), left untreated or treated with NC410 for 48 hours, as indicated in panel **A**. *P ≤ 0.05; **P ≤ 0.01 by 2-tailed Student’s *t* test. (**H**) Flow cytometry histograms of representative donors in **G**, showing both CD206 and CD163 expression with indicated percentage positive cells and MFI (in parentheses) of total cells. Data from RNA-seq analysis are from a single experiment.

Due to the impact of the combination therapy on murine M2 macrophage numbers and transcriptomic profiling in vivo, the effect of LAIR-1, PD-L1, and TGF-β blockade was interrogated in human macrophages polarized in vitro. Adherent cell fractions from peripheral blood mononuclear cells (PBMCs) from healthy donors were cultured for 5 days in the presence of M-CSF, followed by 48-hour polarization as indicated in the schema in Figure 7A. To investigate changes at the RNA level induced by NC410, bintrafusp alfa or NC410 plus bintrafusp alfa on M2-like human macrophages, bulk RNA-seq was conducted. Analysis of the top 500 differentially expressed genes, by variance, across all samples (Figure 7, B and C) indicated that, in this experimental setting, most changes in gene expression were driven by NC410 alone, while the addition of bintrafusp alfa elicited no additional changes (Figure 7D). Interestingly, characterization of the transcriptome of the NC410-treated M2-like macrophages (Figure 7E) revealed significantly decreased expression of genes encoding macrophage polarization markers, including *CD163*, *LAIR1*, and CXCL chemokines (*CXCL1*, *CXCL2*, *CXCL3*, *CXCL5*), and increases in the expression of genes encoding matrix metallopeptidase-12 (*MMP12*), and the chemokines CCL17 and CCL24. In contrast, expression of *MRC1* (CD206), *CD274* (PD-L1), *TGFB1*, and *TGFBR2* remained unchanged. GO analysis showed activation of ECM structure and organization pathways, and inhibition of G2/M checkpoints, E2F targets, and cell division pathways (Figure 7F), which were recently identified to be activated in monocytes and macrophages through LAIR-1 receptor signaling (27). In order to validate some of the observed changes at the protein level, in vitro–polarized macrophages from multiple healthy donors were analyzed via flow cytometry; NC410 treatment of macrophages polarized with IL-4/IL-13/collagen or tumor-conditioned medium (TCM)/collagen consistently and significantly reduced the expression of CD163 on the cell surface (Figure 7G), while not affecting the expression of CD206 (*MRC1*, Figure 7H).

### Collagen, PD-L1, and TGF-β1 are expressed in human colorectal tumors and metastases.

**Figure 8:**
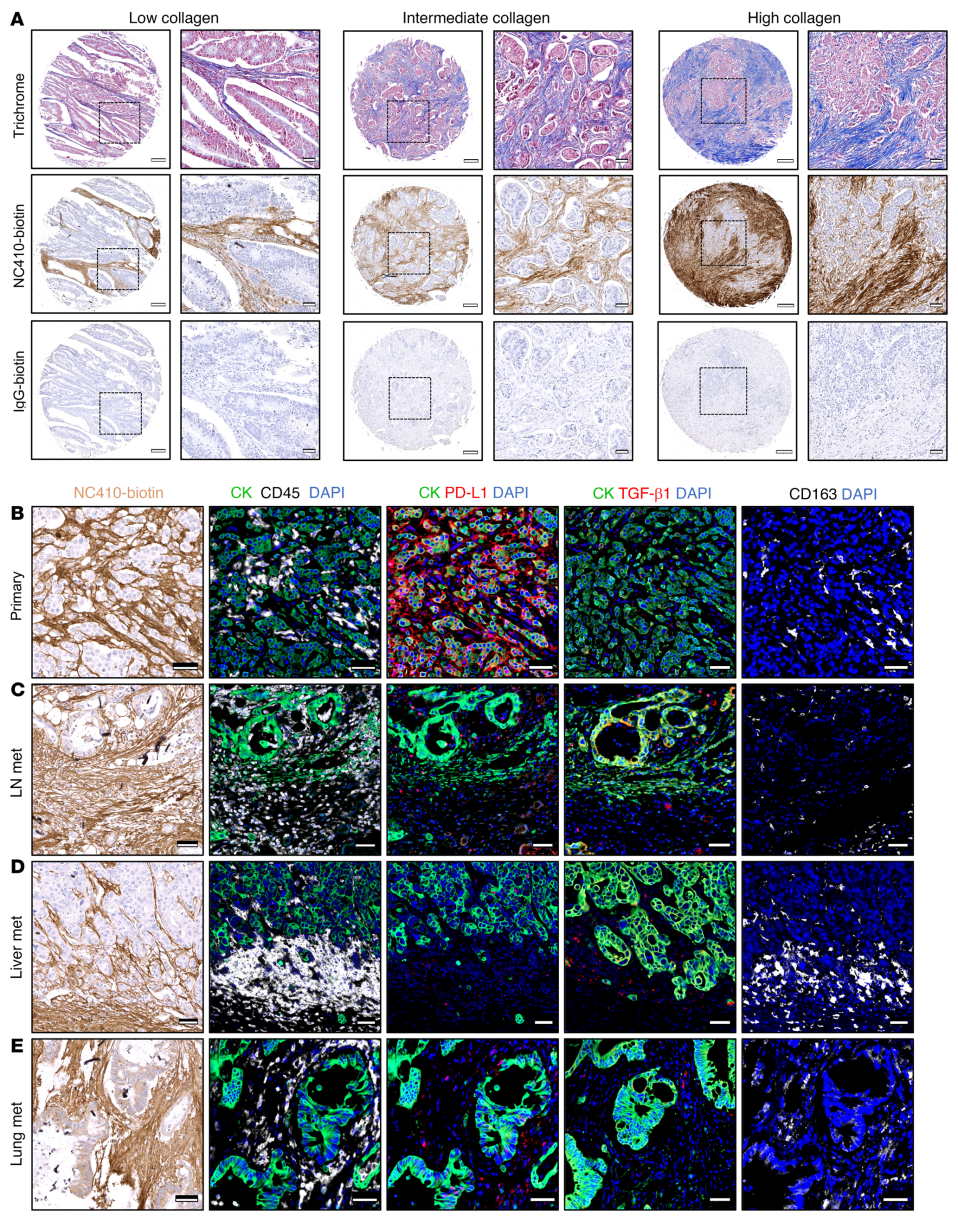
Expression of collagens, PD-L1, and TGF-β1 in colon carcinoma tissues. (**A**) Representative images of tissues from a colon cancer tumor microarray (TMA) stained for collagen content via Masson’s trichrome stain (upper row), NC410-biotin (center row), and control IgG-biotin (bottom row). Shown are a representative case each with low, intermediate, and high collagen content and corresponding low, intermediate, and high binding of NC410. Black dash–outlined squares identify magnified regions in adjacent images. Scale bars: 200 μm (whole sections) and 50 μm (zoomed images). Representative images of (**B**) a primary colon tumor, (**C**) a metastatic lymph node (LN), (**D**) a liver metastasis, and (**E**) a lung metastasis from colon cancer stained for binding of NC410 (NC410-biotin, brown), cytokeratin to identify tumor cells (CK, green), total leukocyte infiltration (CD45, white), PD-L1 (red), CD163 to identify M2-like macrophages (white), and TGF-β1 mRNA by RNA in situ hybridization (red). DAPI was used to stain nuclei (blue). Scale bars: 50 μm (**B** and **C**–**E**).

To determine the level of expression of the targets of the combination immunotherapy and understand its potential relevance to colon cancer treatment, primary and metastatic colon carcinoma tissue microarrays (TMAs) were stained with trichrome, biotinylated NC410, and antibodies directed against PD-L1 or CD163. In addition, expression of TGF-β1 was measured via RNA in situ hybridization. Using trichrome staining, primary colon cancer tissues demonstrated low, intermediate, and high collagen content, which corresponded with the degree and localization of binding of biotinylated NC410, shown for representative examples in Figure 8A. These collagen-rich regions seemingly surrounding tumor cells and tumor cell islands have been proposed to prevent the influx of immune cells into the core of a tumor (28). To evaluate whether collagen-rich tumor areas are characterized by leukocyte trapping, serial sections of primary colon cancer, metastatic lymph nodes, liver, and lung metastatic lesions of colon origin were stained (Figure 8, B–E). Across all primary and metastatic lesions, NC410 binding mostly localized to areas surrounding cytokeratin-positive tumor cells, while also overlapping with areas of CD45^+^ immune cell infiltration. Expression of PD-L1 on tumor cells was observed in a subset of primary tumors and metastatic lesions. PD-L1 was also localized to stroma and immune cells (Figure 8, B–E). Primary and metastatic colon cancer sections showed positive staining for CD163^+^ macrophages; TGF-β1 expression was mostly restricted to stromal and immune cells, with negligible expression in tumor cells. These results provided further support for the combined use of agents targeting collagens, PD-L1, and TGF-β.

## Discussion

This work describes a combinatorial immunotherapy approach consisting of neutralization of PD-L1 and TGF-β with blockade of collagen/LAIR-1 signaling. This combination was able to enhance tumor recruitment and activation of CD8^+^ T cells, reduce M2 macrophage populations, and remodel collagens in the TME, resulting in effective tumor control in murine models, which was not achievable with the individual components of the combination.

While ICB therapy has been revolutionary for the treatment of cancer, as a monotherapy it often fails to provide clinical benefit to the majority of patients due to mechanisms of primary or adaptive resistance (29). These mechanisms cover a range of tumor cell–intrinsic and –extrinsic factors, including loss of antigen presentation, tumor cell plasticity in the context of an epithelial-mesenchymal transition (EMT), recruitment of immunosuppressive cells to the TME, and T cell exclusion (25, 29). TGF-β in particular is a major contributor to tumor T cell exclusion. Analysis of tumors from a large cohort of metastatic urothelial cancer patients, for example, directly linked a TGF-β gene signature to CD8^+^ T cell exclusion from the tumor parenchyma, T cell trapping in the collagen-rich peritumoral stroma, and lack of response to ICB (15). With the idea of overcoming some of these mechanisms of checkpoint resistance, we chose to utilize bintrafusp alfa as one of the components in our combination approach, due to the ability of this agent to block PD-L1 while trapping TGF-β. A clinical-stage agent (23, 30), bintrafusp alfa was shown in preclinical models to reduce TGF-β–mediated tumor EMT (31), enhance the cytolytic ability of T and NK cells, and synergize with other immunotherapies to mediate tumor control (21, 26). Through blockade of TGF-β, this agent was also shown to reduce tumor α-smooth muscle actin (α-SMA) content in tumors, a marker of CAFs, although without affecting the overall collagen content in a murine model of breast cancer (21).

Collagen structure and rigidity inside the TME have been directly linked to physical exclusion of immune cells (32); however, few studies so far have been conducted to evaluate approaches that reduce ECM content or tumor stiffness as a means to increase the efficacy of ICB therapies, especially with tumor types characterized by a collagen-dense TME. One such study, for example, has shown that reducing tumor stiffness and collagen organization via inhibition of the collagen-crosslinking enzyme lysyl oxidase (LOX) increased T cell migration, tumor infiltration with CD8^+^ T cells, and antitumor activity of PD-1 blockade therapy in a model of murine pancreatic cancer (32). In addition to providing a physical obstacle to immune cells, collagens can also bind to the inhibitory receptor LAIR-1, which is expressed across multiple immune cell subsets (33) and correlates with poor prognosis in several tumor types (34, 35). LAIR-1 signaling has been shown in preclinical models to drive T cell suppression and a TIM-3^+^ exhausted T cell phenotype that mediates resistance to ICB (9). To overcome collagen-mediated immunosuppression via LAIR-1, here we utilized the recombinant LAIR-2–IgG fusion protein, NC410 (35), as part of our combination immunotherapy approach. In humanized mouse models, NC410 was recently shown to mediate monotherapy antitumor activity in a T cell–dependent manner, and to localize to collagen-rich areas where LAIR-1^+^ immune cells are located (35). Similarly, using xenograft models in NOD/SCID mice reconstituted with human T lymphocytes, another group showed that a LAIR-2–Fc recombinant protein augmented tumor infiltration with CD8^+^ T cells, resulting in tumor control, and increased the antitumor effect of anti–PD-1 therapy (36). Collectively, these results support the development of a recombinant LAIR-2 fusion protein for potential immunotherapeutic applications, including combinations to augment antitumor activity.

The present work demonstrates that simultaneous blockade of PD-L1, TGF-β, and LAIR-1 exerts optimal antitumor control in the context of collagen-rich tumors. A recent report (37) showed that long-term protection to rechallenge is observed following surgical removal of a primary immunogenic tumor. While both MC38 and EMT6 are considered inherently immunogenic, previous reports have shown that both tumors are only partially responsive to ICB therapies, including bintrafusp alfa (38). Here we show via an ELISPOT assay that the combination therapy markedly and significantly increases the frequency of p15E-specific T cells in the spleens of treated mice above that of monotherapy-treated or untreated mice. We hypothesize that this enhanced frequency of antitumor CD8^+^ T cells plays a central role in the effective control of the primary tumor and may contribute to the long-term tumor protection observed in rechallenge experiments. We show that all 3 components are necessary by enhancing tumor infiltration and activation of CD8^+^ T cells in the tumor parenchyma which, in turn, are indispensable for the efficacy of the combination. Interestingly, blockade of LAIR-1 plus anti–PD-L1 was unable to control the growth of MC38 tumors when treatment was initiated on day 9 after tumor implantation and also failed to promote infiltration or activation of lymphocyte populations under these conditions. We hypothesize that this was due to the presence of active TGF-β signaling in the TME that favored exclusion of immune cells. In support of this idea, combination of LAIR-1 inhibition with a TGF-β trap control agent that blocks TGF-β but lacks binding to PD-L1 did elicit increased tumor infiltration of CD8^+^ T cells and NK cells, although it was to a lesser degree than that observed with the combination NC410 plus bintrafusp alfa. Furthermore, neither the addition of single PD-L1 blockade or TGF-β blockade to LAIR-1 inhibition affected the macrophage compartment in MC38 tumors, an effect that was exclusively observed when all 3 targets (PD-L1, TGF-β, and LAIR-1) were neutralized.

The effect of the combination NC410 plus bintrafusp alfa on tumor macrophages was 2-fold and included the ablation of *Cd163*^pos^ M2 macrophages and the modulation of the transcriptomic profile of the remaining *Cd163*^neg^ M2 population toward a more M1-like phenotype. Due to the point-in-time nature of the scRNA-seq analysis, many questions remain outside of the scope of this current study regarding the mechanism of this phenomenon; however, it was noteworthy that expression of CD163 on human M2-like macrophages polarized in vitro was also significantly downregulated by blockade of LAIR-1 signaling with NC410. The CD163 scavenger receptor is expressed on both murine and human cells of monocytic origin with particularly high expression on M2 macrophages (39). CD163^+^ M2 macrophages are often present in regions of chronic inflammation and have been shown to promote cancer progression and metastases through induction of IL-6 and CXCL2 (40). CD163^+^ M2 macrophages have also been shown to traffic to regions of collagen remodeling, utilizing denatured collagen I as a chemoattractant (41). Denatured or remodeled collagens can arise from the activity of matrix metalloproteinases secreted by tumor, immune, or stromal cells (42). Supporting a potential link between the reduction of *Cd163*^pos^ M2 macrophages and denatured collagens, in this study we found that MC38 tumors treated with NC410 alone or the combination immunotherapy no longer contained denatured collagens, as detected with a CHP that specifically binds to degraded, unfolded collagen chains (43). The lack of denatured collagens could potentially contribute to the loss of the *Cd163*^pos^ M2 population, although further studies are needed to corroborate this link.

With the exception of mismatch repair–deficient (dMMR) tumors, colorectal cancer (CRC) remains recalcitrant to ICB therapy. A study across all molecular subtypes of CRC has demonstrated that a gene program induced by TGF-β in tumor stromal cells can predict poor prognosis across all subtypes (44), leading to the question of whether TGF-β and TGF-β–induced collagens could play a role in CRC resistance to ICB. Here we demonstrate that primary and metastatic colon cancer tissues exhibit a range of collagen content that is bound by NC410, with collagen-rich regions seemingly surrounding epithelial tumor cell islands and overlapping with areas of CD45^+^ immune cell infiltration, supporting the potential exploration of the combination NC410 plus bintrafusp alfa in patients with advanced colon cancer.

This study demonstrates the advantage of combining agents that target immunosuppressive signals derived from the collagenous component of the ECM and the immunosuppressive cytokine TGF-β with classical checkpoint inhibition via anti–PD-L1. The combination approach was able to cure tumors across 2 different murine tumor models in different genetic backgrounds; however, a limitation of this study is the use of s.c. implanted tumors, which may not recapitulate the highly fibrotic stroma of certain human tumors. To overcome this limitation, future studies will be conducted with genetically engineered mouse models that more closely approximate the microenvironment of human tumors. As determined by scRNA-seq analysis and supported with flow cytometry and immunofluorescence-based tissue staining, the combination therapy promoted the infiltration and activation of CD8^+^ T cells in the tumor parenchyma, reshaped and repolarized M2 macrophage phenotypes, and promoted the remodeling of the ECM. This study also provides the rationale for testing of this combination immunotherapy approach in the clinic, potentially in tumor types that do not respond to ICB and are characterized by high levels of collagen, TGF-β, or tumor-infiltrating CD163^+^ M2 macrophages.

## Methods

### Cell lines.

BALB/c-derived breast EMT6 and 4T1 cells, and C57BL/6-derived lung LLC cells were obtained and cultured as recommended by the American Type Culture Collection. The C57BL/6-derived colon MC38 cell line was cultured as previously described (45). Cell lines were determined to be mycoplasma free by using a MycoAlert Mycoplasma Detection Kit (Lonza) and used at low passage number from the date of acquisition.

### Mice.

Female BALB/c and C57BL/6 mice were obtained from the NCI Frederick Cancer Research Facility. Mice were between 5 and 6 weeks old at the start of experiments and were maintained under pathogen-free conditions in accordance with the Association for Assessment and Accreditation of Laboratory Animal Care guidelines.

### Reagents.

Bintrafusp alfa, TGF-β trap control, and anti–PD-L1 were obtained under a Cooperative Research and Development Agreement (CRADA) with EMD Serono. NC410 was obtained under a CRADA with NextCure, Inc.

### Tumor inoculation and treatment schedule.

C57BL/6 mice were inoculated s.c. in the flank with 3 × 10^5^ MC38 cells, and BALB/c mice were inoculated s.c. in the flank with 3 × 10^5^ EMT6 cells. Intraperitoneal injections of bintrafusp alfa (250 μg or 492 μg, as indicated), TGF-β trap control (250 μg), equimolar ratio of anti–PD-L1 (200 μg), and NC410 (125 μg or 250 μg, as indicated) were given on days 9, 11, and 13; animals were sacrificed between 12 and 28 days for analyses. In scRNA-seq experiments, animals received the above doses of agents on days 9 and 11; tumors were collected on day 12. In MC38 flow cytometry experiments, animals received above doses of agents on days 9, 11, and 13; tumors were collected on day 17. For rechallenge experiments, cured and naive mice were injected with the same (3 × 10^5^ MC38 cells for C57BL/6 or 3 × 10^5^ EMT6 for BALB/c) or different (5 × 10^5^ LLC cells for C57BL/6 or 3 × 10^4^ 4T1 cells for BALB/c) tumor cell line at 12 and 28 weeks from the initial tumor injection for MC38 experiments or at 12 and 24 weeks for EMT6 experiments. In all experiments, tumors were measured with a Vernier caliper every 2 to 3 days in 2 perpendicular diameters. Tumor volume = (short diameter^2^ × long diameter)/2.

### Depletion studies.

To deplete immune cells from MC38 tumor–bearing mice, 100 μg of anti-CD4 (clone GK1.5, catalog BP0003-1, BioXcell), 100 μg of anti-CD8 (clone 2.43, catalog BP0061, BioXcell), or 100 μg of anti-NK1.1 (clone PK136, catalog BP0036, BioXcell) depletion antibodies were administered i.p. starting on days 7, 8, and 9 after tumor implantation and then once per week for the duration of the experiment. Intraperitoneal injections of bintrafusp alfa (492 μg) and NC410 (250 μg) were given i.p. on days 8, 10, and 13. Spleens were obtained from animals upon termination of the experiment to determine immune cell population depletion efficiency by flow cytometry (Supplemental Figure 4).

### ELISPOT assay.

C57BL/6 mice bearing MC38 tumors were left untreated or injected with NC410 (250 μg), bintrafusp alfa (492 μg), or a combination of both agents on days 8, 10, and 13. Splenocytes were harvested from mice and assayed ex vivo on day 23 for antigen-dependent cytokine secretion using an IFN-γ ELISPOT assay (BD Biosciences), according to the manufacturer’s instructions. Briefly, 1 × 10^6^ splenocytes were incubated overnight with 10 μg/mL of p15E_604–611_ or a negative control HIV peptide. Antigen-specific cells were quantified using an ImmunoSpot analyzer (Cellular Technology, Ltd). The number of CD8^+^ T cells added per well was calculated by flow cytometry analysis. Data were adjusted to the number of spots/10^4^ CD8^+^ T cells present in the assay, subtracting the number of spots in paired wells containing the control peptide.

### Flow cytometry.

Prior to staining, tumors were weighed, mechanically dissociated, incubated in a shaker at 37°C for 30 minutes in RPMI-1640 medium containing 5% fetal bovine serum (FBS), 5 mg/mL collagenases IV and I (Gibco), and 40 U/mL DNase, and then passed through a 70-μm filter as a single-cell suspension. Spleens were crushed through a 70-μm filter and red cell lysis was performed with ammonium chloride–potassium (ACK) buffer (Gibco). Blood was collected by submandibular bleeding with a lancet into K2EDTA coated Microtainer tubes (BD Biosciences) followed by red cell lysis with ACK. Antibodies used for flow cytometry are listed in Supplemental Table 5. Cells were stained for cell surface expression in flat-bottom 96-well plates on ice in phosphate-buffered saline (PBS) with 2% FBS. Intracellular markers were stained using the eBioscience Foxp3/Transcription Factor Staining Buffer Set according to the manufacturer’s instructions. Fluorescently conjugated antibodies were used as per the manufacturers’ instructions. LIVE/DEAD Fixable Aqua Dead Cell Stain Kit (Thermo Fisher Scientific) was used to gate on live cells. Data were acquired on an Attune NxT Flow Cytometer (Thermo Fisher Scientific) and analyzed via FlowJo (Becton Dickinson). The gating strategy used for analysis is shown in Supplemental Table 6 and Supplemental Figure 5.

### IHC and Masson’s trichrome staining.

NC410 or control human IgG1 (provided by NextCure, Inc.) was biotinylated using the EZ-Link Sulfo-NHS-LC-Biotinylation Kit (Thermo Fisher Scientific) following the manufacturer’s protocol. To evaluate binding to tumor tissue, antigen retrieval was performed via steaming for 30 minutes in pH 9 buffer. Tissues were blocked with BLOXALL Solution (Vector Laboratories) and 2.5% horse serum. Staining was conducted with 1 μg/mL NC410-biotin or IgG-biotin diluted in Renaissance Background Reducing Diluent (BioCare Medical) for 60 minutes at room temperature, followed by streptavidin-HRP and a DAB Substrate Kit (Vector Laboratories). Tissues were counterstained with hematoxylin. Collagen staining was performed on tissues using the Trichrome Stain Kit (Connective Tissue Stain, Abcam) or the Trichrome, McLetchie, Aniline Blue Stain Kit (Newcomer Supply) following the manufacturers’ protocols. In all cases, tissues were rinsed, dehydrated with ethanol and xylene substitute, and mounted in VectaMount mounting medium (Vector Laboratories). Digital images were obtained on a Zeiss Axio Scan.Z1 and Zen Blue software (Zeiss).

### Immunofluorescence.

Antibodies used for immunofluorescence-based detection included anti-CD8a (clone 4SM16, catalog 14-0195-82, Invitrogen), anti–human IgG (polyclonal, catalog SAB3701278, Sigma-Aldrich), anti–wide spectrum cytokeratin (polyclonal, catalog ab9377, Abcam), anti-CD45 (clone HI30, catalog 14-0459-82, Thermo Fisher Scientific), anti–PD-L1 (clone 28.8, catalog ab205921, Abcam), and anti-CD163 (clone 10D6, catalog NB110-59935, Novus). Colon TMAs CO242b (US Biomax) and DCO-702c (US Biolab) were stained in serial sections. Briefly, antigen retrieval was conducted by microwaving in pH 6 buffer (human tissues), or Rodent Decloaker (mouse tissues, Biocare Medical). Slides were rinsed with Tris-buffered saline containing 0.1% Tween (TBST) and blocked with BLOXALL Solution (Vector Laboratories). Staining with primary and secondary antibodies was conducted following the manufacturers’ instructions. For detection, the Opal 4-Color Manual IHC Kit (PerkinElmer) was used.

Unfolded collagens were detected by using a CHP, Cy3 Conjugate (R-CHP, 3Helix Inc.) per the manufacturer’s instructions. Briefly, slides were deparaffinized and rehydrated with xylene substitute and ethanol gradients and either stained immediately with the CHP, pretreated with 1 μg/mL NC410-biotin prior to CHP staining, or microwaved with pH 9 antigen retrieval solution, cooled, and rinsed with TBS prior to CHP staining. The CHP peptide was linearized prior to use by preheating at 80°C for 5 minutes followed by rapid cooling on ice for 60 seconds. As a negative control, non-preheated (nonlinearized) peptide was used. All slides were stained with DAPI (Invitrogen).

Slide scanning and quantification were performed on a Zeiss Axio Scan.Z1 and Zen Blue software. For image quantification, 2 to 5 regions of interest (ROI) were randomly selected per tumor, with no obvious signs of necrosis. Each ROI was analyzed for mean fluorescence intensity (MFI) for the designated marker; when indicated, the MFI was normalized to the signal in the control group.

### RNA in situ hybridization.

TGF-β1 RNA in situ hybridization was performed on tissues using the RNAscope technology (Advanced Cell Diagnostics), following the manufacturer’s protocol. In some experiments, slides were then stained with anti–wide spectrum cytokeratin (ab9377, Abcam). Slide scanning was performed on a Zeiss Axio Scan.Z1 and Zen Blue software.

### In vitro macrophage polarization.

Deidentified PBMC samples were obtained from healthy volunteers who provided written informed consent at the NIH Clinical Center Blood Bank (protocol NCT00001846). PBMCs were plated in DMEM in 12-well tissue culture plates for 2 hours at 37°C. Nonadherent cells were removed and attached cells were cultured for 5 days in RPMI-1640 medium containing macrophage differentiation media: 10% FBS, 1× glutamine, and 50 ng/mL human M-CSF (PeproTech). Media and cytokines were replaced every other day. On day 5, macrophage differentiation media was added with M2-polarizing cytokines: 20 ng/mL IL-4 (PeproTech), 20 ng/mL IL-13 (PeproTech), and 20 μg/mL native mouse collagen I/III (Bio-Rad). Mouse collagen was used due to the highly conserved nature of collagens across species, and the previously demonstrated binding of NC410 to human, mouse, and rat collagen I and III (35). In some experiments MDA-MB-231 TCM plus native mouse collagen I/III (20 μg/mL) were used for M2 polarization. TCM was generated by culturing 3.5 × 10^6^ MDA-MB-231 cells in 20 mL of RPMI-1640 medium plus 10% FBS for 4 days. When indicated, NC410 (100 μg/mL), bintrafusp alfa (200 ng/mL), or the combination was also added. Forty-eight hours after polarization, the attached cells were washed, scraped, and collected for analysis via flow cytometry or processed for bulk RNA-seq.

### Bulk RNA-seq and analysis.

Total RNA from in vitro–polarized macrophages was prepared using the RNeasy Mini Kit (Qiagen). RNA integrity was analyzed on an Agilent TapeStation (Agilent Technologies). Samples with an RNA integrity number (RIN) of greater than 8.0 were sequenced by the Novogene UC Davis Sequencing Center (Novogene Corporation, Ltd.). Briefly, RNA samples were reassessed for integrity using a Nano 6000 Assay Kit for the Bioanalyzer 2100 system (Agilent Technologies); sequence libraries were created using the NEBNext Ultra RNA Library Prep Kit New England Biolabs). Library quality was assessed using the Bioanalyzer 2100, and clustering was carried out utilizing the cBot Cluster Generation System using a PE Cluster Kit cBot-HS (Illumina) according to the manufacturer’s instructions. Library preparations were sequenced, and paired-end reads were generated on an Illumina platform.

Sample reads were processed using the CCBR RNA-seek utility (version 1.2.1). A list of packages and versions used can be found at this URL: https://ccbr.github.io/RNA-seek/RNA-seq/Resources/ Briefly, reads were trimmed for adapters and low-quality bases using Cutadapt (version 1.18) (http://gensoft.pasteur.fr/docs/cutadapt/1.18) before alignment to the human reference genome (hg38/Dec. 2013/GRCh38) from the UCSC browser and the transcripts annotated using STAR v2.7.6a in 2-pass mode (46). Expression levels were quantified using RSEM (version 1.3.3) (47) with GENCODE annotation version 36 (48).

Raw read counts (expected counts from RSEM) were imported to the NIH Integrated Data Analysis Platform (NIDAP) for downstream analysis. Genes with low counts (counts per million [CPM] <0.5) in 3 or more samples were filtered out prior to subsequent analysis. Counts were normalized to library size as CPM and the voom algorithm (49) from the Limma R package (version 3.40.6) (50) was used for quantile normalization. Batch correction was performed prior to analysis using the ComBat function in the sva package (51). Differentially expressed genes using Limma and pathway analysis (GO, KEGG, REACTOME, HALLMARK) of the top 500 up- and downregulated genes by *t* statistic was accomplished by Fisher’s exact test using the l2p package (https://github.com/ccbr/l2p). Genes or gene sets with an adjusted *P* value of 0.05 or less were considered statistically significant.

### scRNA-seq and data analysis.

MC38 tumors obtained 1 day after the second dose of treatment were processed into single-cell suspensions as described above. CD45^+^ cells were enriched using the Miltenyi CD45 (TIL) MicroBeads mouse kit (Miltenyi Biotec). Equal numbers of cells from 2–5 tumors per group (Control, NC410, bintrafusp alfa, NC410 plus bintrafusp alfa, NC410 plus TGF-β trap control, and NC410 plus anti–PD-L1) with greater than 75% cell viability were pooled and used for scRNA-seq using 10× Genomics Cell ranger v4.0.0 at the NCI Single Cell Analysis Facility. Analysis of single-cell data was performed with a standard workflow (Seurat version 3; ref. 52) using a user interface developed in NIDAP as previously described (24). Briefly, cells with greater than 25% mitochondrial expression, or with a number of genes detected above 3 median absolute deviations, were filtered out of downstream analysis. We clustered the data set using Seurat at resolution 2.0. Marker genes for specific cell type identification with module scoring approach were obtained from the literature and are listed in Supplemental Table 1. Genes with greater than 0.25 log(fold change) and *P* value less than 0.05 were considered significantly differentially expressed and used for pathway analysis (GO, KEGG, REACTOME, HALLMARK) using l2p.

### Data and materials availability.

scRNA-seq and bulk RNA-seq data have been deposited in NCBI’s Gene Expression Omnibus (GEO GSE194421 and GSE195685, respectively), and unified under SuperSeries GSE195686.

### Statistics.

All statistical analyses were performed using Prism v.8 for Windows (GraphPad Software). Analysis of tumor growth curves was conducted using 2-way analysis of variance (ANOVA). Statistical differences between 2 sets of data were determined through a 2-tailed Student’s *t* test. One-way ANOVA with Tukey’s post hoc test was used to determine statistical differences among 3 or more sets of data. Error bars represent SEM where noted. Asterisks indicate that the experimental *P* value is statistically significantly different from the associated controls: *P ≤ 0.05, **P ≤ 0.01, ***P ≤ 0.001, ****P ≤ 0.0001.

### Study approval.

All animal studies were approved by the NIH Intramural Animal Care and Use Committee (protocol LTIB-038). Deidentified PBMC samples were obtained from healthy volunteers who provided written informed consent at the NIH Clinical Center Blood Bank (protocol NCT00001846).

## Author contributions

LAH, CP, and JS conceptualized the study. LAH, PLC, SRG, TJM, MC, DF, SL, and CP developed the methodology. LAH, HQ, MI, KF, and CP conducted experiments. LAH, PLC, SRG, TJM, MC, and CP interpreted the data. JS and CP supervised the study. LAH, JS, and CP wrote the manuscript.

## Supplementary Material

Supplemental data

## Version 1 03/01/2022

In-Press Preview

## Version 2 04/15/2022

Electronic publication
