# Supplementary material for: Remodeling the tumor microenvironment via blockade of LAIR-1 and TGF-β signaling enables PD-L1–mediated tumor eradication
Source: J Clin Invest. 2022 Apr 15;132(8):e155148. doi: 10.1172/JCI155148 (PMC9012291; doi:10.1172/JCI155148)
Supplement: Supplemental data [file jci-132-155148-s144.pdf]

A

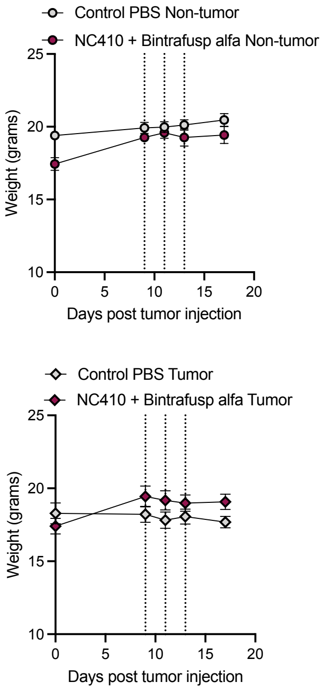

B

	Non-tumor bearing mice					Tumor bearing mice					
	Control PBS		NC410/Bintrafusp alfa			Control PBS			NC410/Bintrafusp alfa		
	Average	SD	Average	SD	*P value**	Average	SD	*P value**	Average	SD	*P value**
WBC Count (K/uL)	1.6	0.9	1.5	0.7	*0.870*	3.12	0.2	*0.101*	1.1	0.2	*0.510*
RBC Count (M/uL)	10.4	0.3	7.9	4.3	*0.287*	5.0	0.1	*<0.001*	11.1	0.4	*0.059*
Hemoglobin g/dL	15.1	0.5	11.5	6.0	*0.279*	7.7	0.1	*<0.001*	15.8	0.6	*0.147*
Hematocrit %	52.2	1.9	40.5	21.9	*0.327*	28.6	2.1	*<0.001*	56.4	2.5	*0.078*
MCV fL	50.3	1.1	51.8	0.8	*0.079*	57.9	5.0	*0.032*	51.0	0.2	*0.507*
Platelets K/uL	858.3	241.8	511.3	411.0	*0.196*	823.0	53.7	*0.857*	746.0	138.6	*0.588*
Polys %	10.0	2.3	11.4	3.4	*0.521*	27.9	2.5	*0.001*	14.6	1.2	*0.062*
Lymphocytes %	79.0	6.2	72.8	10.5	*0.346*	65.5	2.6	*0.047*	73.9	0.6	*0.329*
Monocytes %	1.2	0.3	2.7	2.1	*0.202*	1.5	0.8	*0.534*	2.9	0.5	*0.006*
Eosinophils %	8.8	4.5	10.7	8.6	*0.701*	2.2	0.1	*0.122*	7.7	0.8	*0.758*
Basophils %	0.4	0.1	1.1	0.8	*0.140*	0.4	0.2	*0.674*	0.5	0.1	*0.506*
WBC Count %	1.6	0.9	1.5	0.7	*0.870*	3.1	0.2	*0.101*	1.1	0.2	*0.510*

(*) P values compared to control PBS group

C

	Non-tumor bearing mice					Tumor bearing mice					
	Control PBS		NC410/Bintrafusp alfa			Control PBS			NC410/Bintrafusp alfa		
	Average	SD	Average	SD	*P value**	Average	SD	*P value**	Average	SD	*P value**
Alkaline Phos (U/L)	191.0	18.6	140.0	65.5	*0.185*	69.3	13.6	*<0.001*	145.7	67.3	*0.244*
ALT/GPT (U/L)	38.3	4.7	93.5	109.8	*0.354*	26.0	2.0	*0.009*	27.0	3.0	*0.016*
Cholesterol (mg/dL)	85.0	8.8	89.8	11.4	*0.534*	91.0	6.1	*0.360*	90.3	2.3	*0.360*
Triglycerides (mg/dL)	84.0	7.5	116.3	24.8	*0.047*	136.7	57.3	*0.119*	75.5	30.4	*0.585*
Sodium (mmol/L)	148.8	1.3	112.2	69.1	*0.330*	149.3	0.6	*0.496*	148.3	2.1	*0.753*
Potassium (mmol/L)	7.6	0.3	33.6	50.9	*0.345*	6.4	0.4	*0.006*	7.3	0.8	*0.624*
Chloride (mmol/L)	110.5	1.7	108.3	2.1	*0.192*	113.0	1.7	*0.117*	112.3	1.5	*0.206*
Albumin (g/dL)	4.4	0.1	4.3	0.1	*0.207*	3.6	0.2	*<0.001*	4.1	0.5	*0.377*
CK, Total (U/L)	208.7	142.0	4316.0	2727.1	*0.060*	342.0	297.8	*0.523*	239.0	113.0	*0.787*
LD (U/L)	249.8	159.2	564.5	240.7	*0.072*	409.3	128.3	*0.216*	208.0	4.2	*0.744*
Protein, Total (g/dL)	5.6	0.1	5.7	0.2	*0.390*	4.8	0.2	*0.001*	5.3	0.5	*0.409*
Uric Acid (mg/dL)	1.4	0.6	1.6	0.5	*0.601*	0.8	0.3	*0.152*	0.6	0.0	*0.137*

(*) P values compared to control PBS group

D

Organ	Non-tumor bearing mice		Tumor bearing mice	
	Control PBS	NC410/Bintrafusp alfa	Control PBS	NC410/Bintrafusp alfa
Brain	5/5 normal	5/5 normal	5/5 normal	5/5 normal
Heart	5/5 normal	5/5 normal	4/5 normal<br>1/5 mild chronic myocarditis	5/5 normal
Kidney	2/5 normal<br>3/5 mild chronic ureteritis	2/5 normal<br>1/5 mild chronic perinephritis<br>2/5 mild chronic ureteritis	5/5 normal	4/5 normal<br>1/5 mild chronic ureteritis
Liver	5/5 mild chronic active hepatitis	5/5 mild chronic active hepatitis	2/5 normal<br>3/5 mild chronic active hepatitis	5/5 mild chronic active hepatitis

Supplemental Figure 1. Murine toxicity analysis. MC38 tumor-bearing and non-tumor C57BL/6 mice were administered PBS or 250µg NC410 plus 492µg bintrafusp alfa on days 9, 11, and 13 post-tumor injection (n=5 mice/group). (A) Change in body weight over time. Vertical lines indicate drug administration dates; error bars indicate SEM of biological replicates. (B) On day 17, mice were sacrificed to evaluate (B) blood CBC counts, (C) serum chemistry, and (D) pathological differences in major organs (brain, heart, kidney, liver). Tissues were examined by a board-certified pathologist (VitroVivo Biotech); blood and serum samples were analyzed by the Department of Laboratory Medicine, NIH Clinical Center.

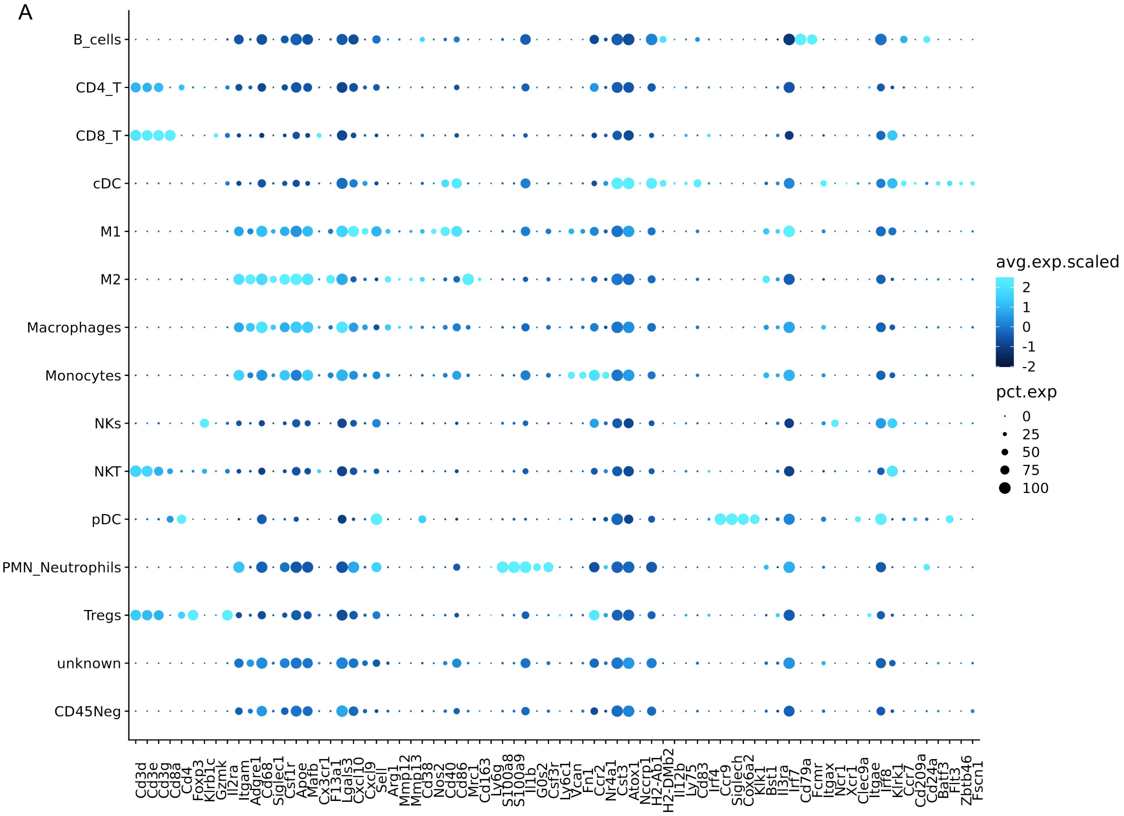

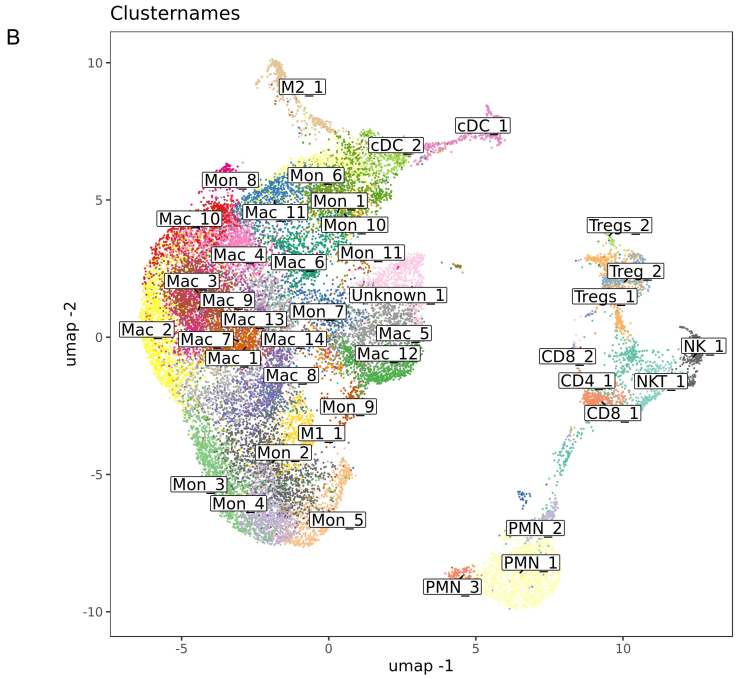

Supplemental Figure 2. Identification of immune cell subsets. (A) Bubble plot depicting expression of genes used to identify cell types across all immune cell subsets. (B) UMAP of identified immune cell subset clusters. Each dot represents one cell and is colored according to cell clusters. Clusters were named according to the most abundant major immune cell subset identified in the cluster.

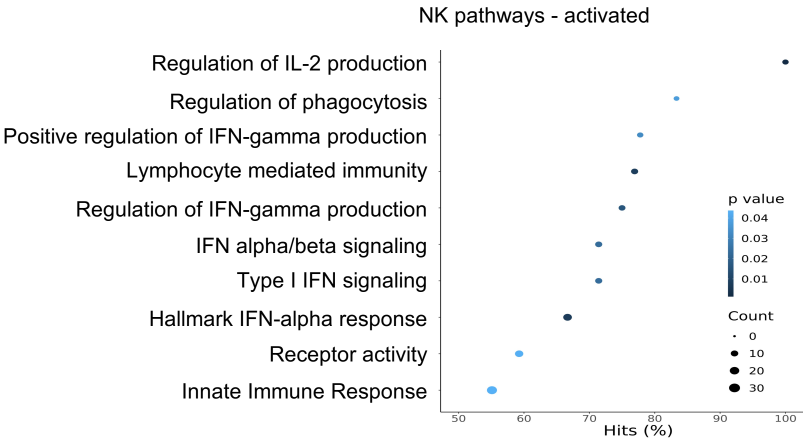

Supplemental Figure 3. Selected activated GO/REACTOME/KEGG/HALLMARK gene pathways in NK cell clusters identified by scRNAseq in the NC410 plus bintrafusp alfa vs. the control group.

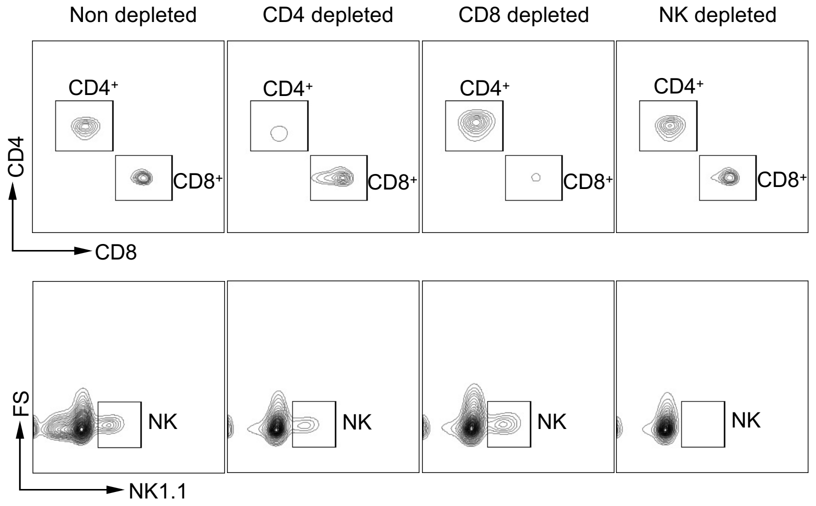

Supplemental Figure 4. Scatter plots demonstrate CD4+, CD8+, and NK-cell depletion efficiency in spleens of representative mice from Figure 3G.

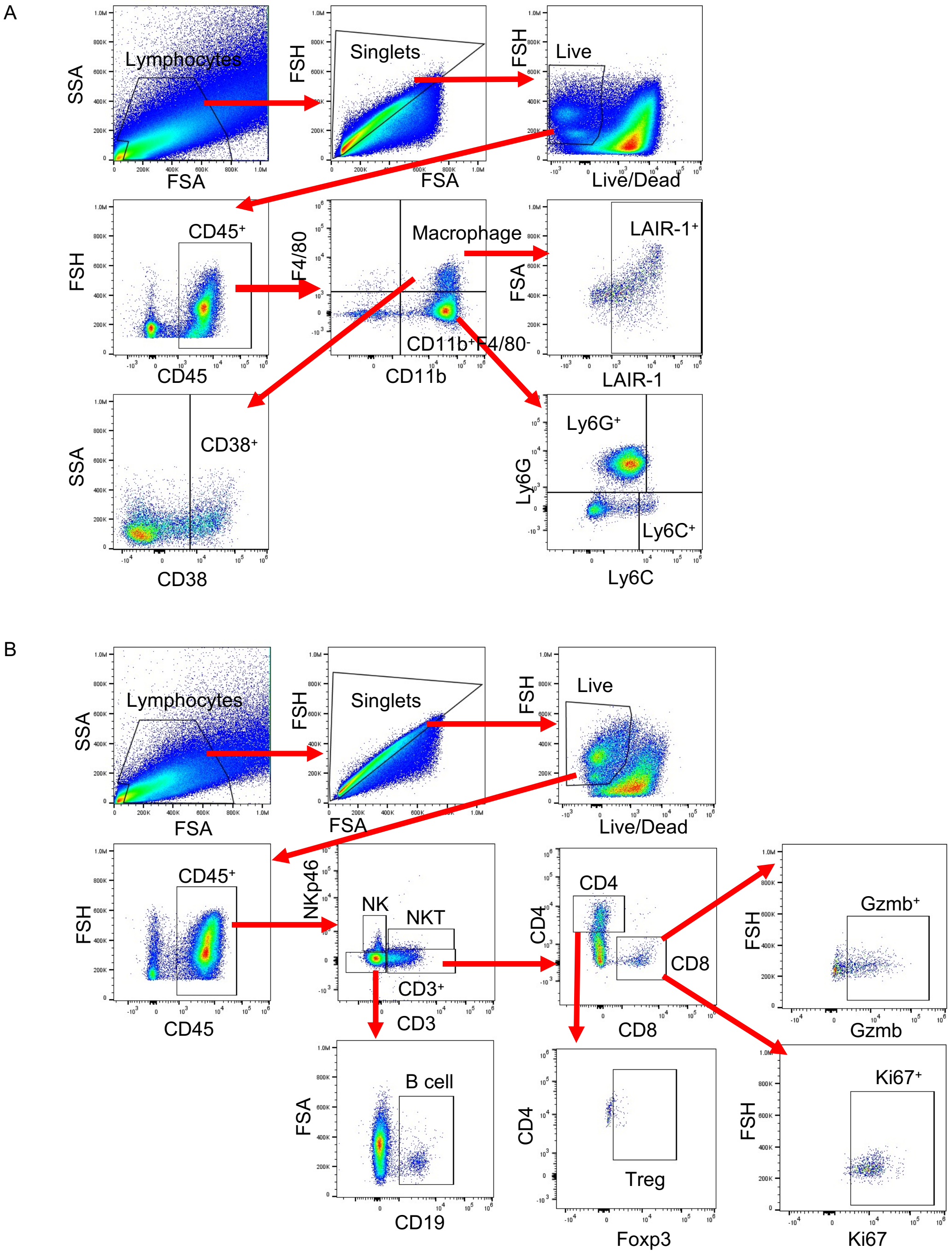

Supplemental Figure 5. Flow cytometry gating strategy. (A) Gating strategy used to identify macrophages and their subsequent phenotype, CD11b+Ly6G+ cells, CD11b+Ly6c+ cells. (B) Gating strategy used to identify NK cells, NKT cells, CD4 T cells, Tregs, CD8 T cells and their subsequent phenotypes, and B cells.

Supplemental Table 1. Genes used to identify murine immune cell subtypes by scRNAseq.

CD8 T	CD4 T	Treg	Macrophage	M1	M2	Neutrophil	Monocyte	cDC	pDC	B cell	NK	NKT
Cd3e pos	*Cd3e pos*	*Cd3e pos*	*Itgam high*	*Cd38 pos*	*Cd38 neg*	*Ly6g pos*	*Itgam pos*	*Cst3 pos*	*Ccr9 pos*	*Cd79a pos*	*Cd3e neg*	*Cd3e pos*
Cd8a pos	*Cd4 pos*	*Cd4 pos*	*Ly6g neg*	*Mrc1 neg*	*Mrc1 pos*	*Ly6c1 low*	*Ly6c1 pos*	*Atox pos*	*Siglech pos*	*Fcmr pos*	*Cd3d neg*	*Cd3d pos*
Cd3d pos	*Foxp3 neg*	*Foxp3 pos*	*Adgre1 pos*	*Nos2 pos*	*Cd163 pos*	*Adgre1 neg*	*Ly6g low*	*Nccrp1 pos*	*Cox6a2 pos*		*Ncr1 pos*	*Klrb1c pos*
Cd3g pos	*Cd3d pos*	*Cd3d pos*	*Cd68 pos*	*Sell low*		*S100a9 pos*	*Vcan pos*	*H2-Ab1 pos*	*Klk1 pos*		*Klrb1c pos*	*Klrk1 pos*
Cd4 neg	*Cd3g pos*	*Cd8a neg*	*Siglec1 pos*	*Cd40 pos*		*S100a8 pos*	*Fn1 pos*	*H2-Dmb2 pos*	*Bst1 pos*			*Ncr1 neg*
Foxp3 neg	*Cd8a neg*	*Il2ra pos*	*Csf1r pos*	*Cd86 pos*		*Il1b pos*	*Ccr2 pos*	*Itgax pos*	*Il3ra pos*			
Gzmk pos	*Klrb1c neg*	*Klrb1c neg*	*Apoe pos*			*G0s2 pos*	*Csf1r pos*	*Il12b pos*	*Irf7 pos*			
Klrb1c neg			*Mafb pos*			*Csf3r pos*	*Mafb high*	*Xcr1 pos*				
Klrk1 pos			*Cx3cr1 pos*				*Nr4a1 pos*	*Clec9a pos*				
			Nr4a1 neg				*Lgals3 pos*	*Itgae pos*				
			F13a1 pos					*Irf8 pos*				
			Lgals3 pos					*Klrk1 pos*				
			Cxcl9 pos					*Cccr7 pos*				
			Cxcl10 pos					*Cd209a pos*				
			Sell low					*Cd24a pos*				
			Arg1 pos					*Batf3 pos*				
			Mmp13 pos					*Flt3 pos*				
			Mmp12 pos					*Zbtb46 pos*				
								Fscn1 pos				
								Ly75 pos				
								Cd83 pos				
								Adgre1 neg				
								Irf4 pos				

Supplemental Table 2. Number of cells identified in each immune cell subset across treatment groups.

Cell subtype	*Control*	*NC410*	*Bintrafusp alfa*	*NC410 + Bintrafusp alfa*	*NC410 + Mutant*	*NC410 + anti-PD-L1*
CD8	73	85	93	283	184	66
CD4	44	64	62	77	58	29
Treg	68	107	139	227	151	62
NK	31	61	24	98	99	27
NKT	15	21	13	56	33	7
Macrophage	1432	1258	1972	1578	1376	934
M1	227	230	345	327	273	247
M2	80	53	132	69	50	64
Monocyte	1048	1222	1889	1294	1783	869
PMN	211	216	294	180	275	213
cDC	156	195	207	131	207	120
B cell	5	5	5	1	6	0
pDC	3	2	1	6	4	6
CD45-	93	121	142	66	119	78
Unknown	568	793	717	456	678	424
Total	4054	4433	6035	4849	5296	3146

Supplemental Table 3. NK activated pathways. List of all GO/KEGG/REACTOME/HALLMARK pathways from upregulated genes in NK cells from NC410 plus bintrafusp alfa-treated vs. control.

Pathway Name	***Category***	***Pathway Accession Identifier***	***P value***
regulation of interleukin-2 production	GO	GO:0032663	0.001507197
protein binding, bridging	GO	GO:0030674	0.003767993
binding, bridging	GO	GO:0060090	0.003767993
HALLMARK_INTERFERON_ALPHA_RESPONSE	H	M5911	0.007335639
lymphocyte mediated immunity	GO	GO:0002449	0.008275381
adaptive immune response based on somatic recombination of immune receptors built from immunoglobulin superfamily domains	GO	GO:0002460	0.008680388
defense response to virus	GO	GO:0051607	0.00871356
humoral immune response mediated by circulating immunoglobulin	GO	GO:0002455	0.009456923
complement activation	GO	GO:0006956	0.009456923
complement activation, classical pathway	GO	GO:0006958	0.009456923
response to interferon-alpha	GO	GO:0035455	0.009456923
protein activation cascade	GO	GO:0072376	0.009456923
Regulation of TP53 Activity	REACTOME	R-HSA-5633007	0.009456923
humoral immune response	GO	GO:0006959	0.01296944
regulation of interferon-gamma production	GO	GO:0032649	0.015927266
defense response to other organism	GO	GO:0098542	0.017014228
chromatin remodeling	GO	GO:0006338	0.017332767
immunoglobulin mediated immune response	GO	GO:0016064	0.017332767
B cell mediated immunity	GO	GO:0019724	0.017332767
protein localization to nucleus	GO	GO:0034504	0.017332767
blood microparticle	GO	GO:0072562	0.017332767
regulation of protein catabolic process	GO	GO:0042176	0.023237194
type I interferon signaling pathway	GO	GO:0060337	0.023237194
Interferon alpha/beta signaling	REACTOME	R-HSA-909733	0.023237194
calcium ion binding	GO	GO:0005509	0.030188002
lipid localization	GO	GO:0010876	0.030188002
positive regulation of interferon-gamma production	GO	GO:0032729	0.032723879
ameboidal-type cell migration	GO	GO:0001667	0.037901575
mRNA 3'-UTR binding	GO	GO:0003730	0.037901575
response to interferon-beta	GO	GO:0035456	0.037901575
regulation of phagocytosis	GO	GO:0050764	0.037901575
positive regulation of phagocytosis	GO	GO:0050766	0.037901575
modulation of synaptic transmission	GO	GO:0050804	0.037901575
Generic Transcription Pathway	REACTOME	R-HSA-212436	0.041421542
response to bacterium	GO	GO:0009617	0.042199234
receptor activity	GO	GO:0004872	0.043066745
innate immune response	GO	GO:0045087	0.043637202
multi-organism reproductive process	GO	GO:0044703	0.047802405
defense response	GO	GO:0006952	0.049411404

Supplemental Table 4. M2 activated pathways. List of all GO/KEGG/REACTOME/HALLMARK pathways from upregulated genes in $Cd163^{neg}Mrc1^{pos}$ cells from NC410 plus bintrafusp alfa-treated vs. control.

Pathway Name	*Category*	*Pathway Accession Identifier*	*P value*
HALLMARK_INTERFERON_GAMMA_RESPONSE	H	M5913	1.23E-07
HALLMARK_INTERFERON_ALPHA_RESPONSE	H	M5911	7.25E-06
defense response to virus	GO	GO:0051607	1.34E-05
response to virus	GO	GO:0009615	5.83E-05
regulation of innate immune response	GO	GO:0045088	1.34E-04
response to cytokine	GO	GO:0034097	1.78E-04
positive regulation of defense response	GO	GO:0031349	2.77E-04
lymphocyte activation	GO	GO:0046649	3.10E-04
cytokine-mediated signaling pathway	GO	GO:0019221	3.14E-04
cellular response to cytokine stimulus	GO	GO:0071345	4.05E-04
positive regulation of innate immune response	GO	GO:0045089	5.39E-04
interferon signaling	REACTOME	R-HSA-913531	5.39E-04
defense response to other organism	GO	GO:0098542	9.01E-04
activation of innate immune response	GO	GO:0002218	9.29E-04
innate immune response-activating signal transduction	GO	GO:0002758	9.29E-04
response to interferon-gamma	GO	GO:0034341	9.29E-04
response to type I interferon	GO	GO:0034340	0.001669373
Interferon alpha/beta signaling	REACTOME	R-HSA-909733	0.001669373
protein polyubiquitination	GO	GO:0000209	0.002570106
lymphocyte activation involved in immune response	GO	GO:0002285	0.002570106
Interleukin-10 signaling	REACTOME	R-HSA-6783783	0.002570106
HALLMARK_ALLOGRAFT_REJECTION	H	M5950	0.002701157
chromatin organization	GO	GO:0006325	0.002977242
type I interferon signaling pathway	GO	GO:0060337	0.002977242
cytokine signaling in immune system	REACTOME	R-HSA-1280215	0.00352887
RNA binding	GO	GO:0003723	0.004446469
positive regulation of cell-cell adhesion	GO	GO:0022409	0.005267284
positive regulation of leukocyte cell-cell adhesion	GO	GO:1903039	0.005267284
regulation of mononuclear cell migration	GO	GO:0071675	0.005374827
regulation of lymphocyte migration	GO	GO:2000401	0.005374827
chemokine receptors bind chemokines	REACTOME	R-HSA-380108	0.005374827
immune response-activating signal transduction	GO	GO:0002757	0.005610886
immune response-regulating signaling pathway	GO	GO:0002764	0.007484486
histone binding	GO	GO:0042393	0.008368472
B-cell activation	GO	GO:0042113	0.008368472
negative regulation of viral genome replication	GO	GO:0045071	0.008368472
chemokine-mediated signaling pathway	GO	GO:0070098	0.008368472
G alpha (i) signalling events	REACTOME	R-HSA-418594	0.008368472
HALLMARK_MITOTIC_SPINDLE	H	M5893	0.008368472
viral process	GO	GO:0016032	0.009126287
covalent chromatin modification	GO	GO:0016569	0.009234611
positive regulation of T cell activation	GO	GO:0050870	0.009234611
pattern recognition receptor signaling pathway	GO	GO:0002221	0.009369309
negative regulation of viral life cycle	GO	GO:1903901	0.009369309
actin cytoskeleton reorganization	GO	GO:0031532	0.011264741
mononuclear cell proliferation	GO	GO:0032943	0.011264741
lymphocyte proliferation	GO	GO:0046651	0.011264741
leukocyte proliferation	GO	GO:0070661	0.011264741
positive regulation of mononuclear cell migration	GO	GO:0071677	0.011264741
regulation of monocyte chemotaxis	GO	GO:0090025	0.011264741
positive regulation of lymphocyte migration	GO	GO:2000403	0.011264741
regulation of lymphocyte chemotaxis	GO	GO:1901623	0.011264741
multi-organism cellular process	GO	GO:0044764	0.012001685
chemokine signaling pathway	KEGG	ko04062	0.012518823
chromosome organization	GO	GO:0051276	0.012518823
nucleolus	GO	GO:0005730	0.014800001
regulation of leukocyte activation	GO	GO:0002694	0.015835108
immunological synapse	GO	GO:0001772	0.01600751
regulation of protein polymerization	GO	GO:0032271	0.01600751
positive regulation of tumor necrosis factor superfamily cytokine production	GO	GO:1903557	0.01600751
factors involved in megakaryocyte development and platelet production	REACTOME	R-HSA-983231	0.01600751
negative regulation of viral process	GO	GO:0048525	0.016024768
cellular response to interferon-gamma	GO	GO:0071346	0.016024768
class A/1 (rhodopsin-like receptors)	REACTOME	R-HSA-373076	0.016915714
regulation of T cell activation	GO	GO:0050863	0.017137306
regulation of cell activation	GO	GO:0050865	0.018132362
regulation of cell-cell adhesion	GO	GO:0022407	0.018199046
innate immune response	GO	GO:0045087	0.018799982
symbiosis, encompassing mutualism through parasitism	GO	GO:0044403	0.018942238
interspecies interaction between organisms	GO	GO:0044419	0.018942238
single-organism organelle organization	GO	GO:1902589	0.018942238
positive regulation of immune system process	GO	GO:0002684	0.020313971
positive regulation of response to external stimulus	GO	GO:0032103	0.021443905
positive regulation of cell adhesion	GO	GO:0045785	0.022642859
response to biotic stimulus	GO	GO:0009607	0.023141316
response to other organism	GO	GO:0051707	0.023141316
response to external biotic stimulus	GO	GO:0043207	0.023141316
lymphocyte homeostasis	GO	GO:0002260	0.023660669
DNA repair	GO	GO:0006281	0.023660669
carbohydrate catabolic process	GO	GO:0016052	0.023660669
lymphocyte costimulation	GO	GO:0031294	0.023660669
T cell costimulation	GO	GO:0031295	0.023660669
negative regulation of type I interferon production	GO	GO:0032480	0.023660669
regulation of erythrocyte differentiation	GO	GO:0045646	0.023660669
regulation of dendrite morphogenesis	GO	GO:0048814	0.023660669
positive regulation of monocyte chemotaxis	GO	GO:0090026	0.023660669
dendritic cell migration	GO	GO:0036336	0.023660669
single-organism carbohydrate catabolic process	GO	GO:0044724	0.023660669
prolactin signaling pathway	KEGG	hsa04917	0.023660669
TRAF6 mediated IRF7 activation	REACTOME	R-HSA-933541	0.023660669
Herpes simplex infection	KEGG	ko05168	0.023971088
positive regulation of hemopoiesis	GO	GO:1903708	0.023971088
immune system development	GO	GO:0002520	0.024961314
DNA metabolic process	GO	GO:0006259	0.027485534
lymphocyte differentiation	GO	GO:0030098	0.027485534
regulation of cell morphogenesis	GO	GO:0022604	0.02787926
regulation of leukocyte cell-cell adhesion	GO	GO:1903037	0.02787926
immune response	GO	GO:0006955	0.028398012
toll-like receptor signaling pathway	KEGG	ko04620	0.030217242
toll-like receptor signaling pathway	GO	GO:0002224	0.030217242
positive regulation of homeostatic process	GO	GO:0032846	0.030217242
Hepatitis B	KEGG	hsa05161	0.030217242
cell adhesion molecules (CAMs)	KEGG	ko04514	0.030392198
JAK-STAT cascade	GO	GO:0007259	0.030392198
regulation of sequestering of calcium ion	GO	GO:0051282	0.030392198
negative regulation of leukocyte activation	GO	GO:0002695	0.032880824
positive regulation of cell activation	GO	GO:0050867	0.033820627
nucleic acid binding	GO	GO:0003676	0.036703699
organelle organization	GO	GO:0006996	0.038403466
immune system	REACTOME	R-HSA-168256	0.038528527
positive regulation of cytosolic calcium ion concentration	GO	GO:0007204	0.03942056
modification of morphology or physiology of other organism	GO	GO:0035821	0.040875003
regulation of lymphocyte activation	GO	GO:0051249	0.040875003
modification of morphology or physiology of other organism involved in symbiotic interaction	GO	GO:0051817	0.040875003
immune system process	GO	GO:0002376	0.040878032
negative regulation of multi-organism process	GO	GO:0043901	0.042474673
regulation of cell shape	GO	GO:0008360	0.046520509
regulation of type I interferon production	GO	GO:0032479	0.046520509
positive regulation of leukocyte activation	GO	GO:0002696	0.049937585
positive regulation of lymphocyte activation	GO	GO:0051251	0.049937585

Supplemental Table 5. Antibodies used in flow cytometry panels.

Marker	*Clone*	*Catalog Number*	*Dilution*	*Manufacturer*
**CD3e**	500A2	152316	1:100	Biolegend
**CD4**	L3T4	100447	1:100	Biolegend
**CD8a**	53-6.7	100750	1:100	Biolegend
**CD11b**	M1/70	101212	1:100	Biolegend
**CD19**	1D3/CD19	152410	1:100	Biolegend
**CD38**	90	102732	1:100	Biolegend
**CD45**	30-F11	103116	1:100	Biolegend
**F4/80**	Bm8	123147	1:100	Biolegend
**Foxp3**	150D	320012	1:20	Biolegend
**Gzmb**	QA18A28	396406100	1:20	Biolegend
**Ki67**	16A8	652406	1:20	Biolegend
**Lair1**	113	12-3051-82	1:100	ThermoFisher
**Ly6C**	HK1.4	128012	1:100	Biolegend
**Ly6G**	1A8	127645	1:100	Biolegend
**NKp46**	29A1.4	137619	1:100	Biolegend
**NK1.1**	PK136	557391	1:100	BD Biosciences
**CD68**	Y1/82A	333814	1:25	Biolegend
**CD163**	GHI/61	333618	1:25	Biolegend
**CD206**	19.2	12-2069-42	1:25	eBioscience

Supplemental Table 6. Flow cytometry gating strategy for major murine immune cell subtypes.

Cell Type	***Gating Strategy***
$CD8^{+}$ T cells	Singlets>Live/Dead Dye^{neg}>$CD45^{+}$>$CD3^{+}$>$CD8^{+}$
$CD4^{+}$ T cells	Singlets>Live/Dead Dye^{neg}>$CD45^{+}$>$CD3^{+}$>$CD4^{+}$
Tregs	Singlets>Live/Dead Dye^{neg}>$CD45^{+}$>$CD4^{+}$>$FoxP3^{+}$
Macrophages	Singlets>Live/Dead Dye^{neg}>$CD45^{+}$>$CD11b^{+}$>$F4/80^{hi}$
$CD11b^{+}Ly6G^{+}$	Singlets>Live/Dead Dye^{neg}>$CD45^{+}$>$CD11^{+}$>$F4/80^{neg/lo}$>$Ly6G^{+}$
$CD11b^{+}Ly6C^{+}$	Singlets>Live/Dead Dye^{neg}>$CD45^{+}$>$CD11b^{+}$>$F4/80^{neg/lo}$>$Ly6G^{neg}$>$Ly6C^{hi}$
$CD19^{+}$	Singlets>Live/Dead Dye^{neg}>$CD45^{+}$>$CD3^{neg}$>$CD19^{+}$
NK	Singlets>Live/Dead Dye^{neg}>$CD45^{+}$>$CD3^{neg}$>$NKp46^{+}$
NKT	Singlets>Live/Dead Dye^{neg}>$CD45^{+}$>$CD3^{+}$>$NKp46^{+}$
All $CD11b^{+}$	Singlets>Live/Dead Dye^{neg}>$CD45^{+}$>$CD11b^{+}$
